# Selective Neuronal Vulnerability to Alpha-Synuclein Pathology in Parkinson’s Disease: A Critical Review of Mechanistic Rationale and Biomarker Stratification

**DOI:** 10.3390/medsci14040489

**Published:** 2026-08-17

**Authors:** Livia Livinț-Popa, Andreea Nicolaie, Alexandra Mastaleru, Ștefan Socolov, Mihaela Mitrea, Gabriela Popescu, Thomas Gabriel Schreiner, Roxana Covali, Laura-Elena Cucu, Lucia Corina Dima-Cozma, Romica Sebastian Cozma, Alin Ciubotaru, Raluca Olariu, Cosmin Mihai Ciurumelea, Liana Rada Borza, Albert Vamanu

**Affiliations:** 1 Department of Neuroscience, “Iuliu Hațieganu” University of Medicine and Pharmacy, 400083 Cluj-Napoca, Romania; livia.popa@umfcluj.ro; 2 American University of the Caribbean School of Medicine, Miramar, FL 33025, USA; andreeanicolaie@students.aucmed.edu; 3 Grigore T. Popa University of Medicine and Pharmacy, 700115 Iasi, Romania; alexandra.mastaleru@umfiasi.ro (A.M.); socolov.stefan9@gmail.com (Ș.S.); mihaela.mitrea77@gmail.com (M.M.); gabriela.popescu@umfiasi.ro (G.P.); cozma.dima@umfiasi.ro (L.C.D.-C.); sebastian.cozma@umfiasi.ro (R.S.C.); alinciubotaru94@yahoo.com (A.C.); raluca.olariu@umfiasi.ro (R.O.); 4 Prevent Institute, 020021 Bucharest, Romania; ciurumeleacosminmihai@yahoo.ro; 5 Faculty of Medicine, Imperial College London, London SW7 2AZ, UK; clinicapsihoger@yahoo.com; 6 Basic and Clinical Neuroscience Department, Institute of Psychiatry, Psychology and Neuroscience, King’s College London, London SE5 8AF, UK; albert.vamanu@kcl.ac.uk

**Keywords:** Parkinson’s disease, selective neuronal vulnerability, alpha-synuclein, neurodegeneration, lysosomal dysfunction, GBA1, glucocerebrosidase, mitochondrial dysfunction, neuroinflammation, biomarkers, precision medicine, Parkinson’s Vulnerability Index

## Abstract

**Background:** Parkinson’s disease (PD) exhibits remarkable clinical heterogeneity, with substantial variability in motor progression, cognitive decline, and the development of non-motor manifestations. Although the propagation of pathological alpha-synuclein is considered a central mechanism of PD pathogenesis, it does not fully explain why specific neuronal populations demonstrate differential susceptibility to degeneration. Emerging evidence suggests that selective neuronal vulnerability is determined by the interaction of multiple biological domains, including calcium homeostasis, mitochondrial bioenergetic capacity, lysosomal function, axonal architecture, synaptic resilience, and neuroinflammatory responses. **Methods:** A structured critical narrative review was performed using PubMed/MEDLINE, Scopus, and Web of Science databases from inception to March 2025. Only full-text articles published in English and peer-reviewed journals were included. Evidence from human post-mortem studies, genetic analyses, biomarker investigations, experimental models, induced pluripotent stem cell studies, and longitudinal clinical cohorts were systematically synthesised to identify the principal mechanisms underlying selective neuronal vulnerability and their potential translational application. To capture evidence published after this electronic cut-off, a supplementary manual review of reference lists of key systematic reviews and landmark publications was conducted up to the date of manuscript submission; references with 2025 or 2026 publication dates entered through this supplementary process. The supplementary manual review was conducted as a targeted scan of reference lists of key systematic reviews and high-impact publications identified during the primary search and did not constitute an independent updated systematic search. **Results:** Five interconnected biological domains emerged as key determinants of neuronal susceptibility in PD: calcium-mediated metabolic stress associated with autonomous pacemaker activity, mitochondrial dysfunction and energetic failure, impaired lysosomal degradation and proteostasis (particularly involving the GBA1–glucocerebrosidase pathway), vulnerability related to extensive axonal and synaptic architecture, and neuroinflammatory mechanisms involving microglial and astrocytic activation. Among these domains, the lysosomal pathway currently provides the strongest translational link between molecular mechanisms and measurable clinical outcomes. Based on this integrated framework, we propose the Parkinson’s Vulnerability Index (PVI), a hypothesis-generating multidimensional model combining genetic, enzymatic, alpha-synuclein seeding, cognitive, olfactory, and neuroimaging biomarkers to facilitate biological stratification and improve the design of mechanism-targeted clinical trials. **Conclusions:** Selective neuronal vulnerability provides a complementary framework to alpha-synuclein propagation models for understanding the heterogeneity of PD. The proposed PVI is not intended as a diagnostic or prognostic clinical instrument but as a research tool requiring prospective validation. Future precision medicine approaches in PD may benefit from integrating vulnerability-related biomarkers with existing biological staging systems to identify patients most likely to benefit from targeted disease-modifying therapies.

## 1. Introduction

### 1.1. Parkinson’s Disease: From Pathological Spread to Clinical Heterogeneity

Parkinson’s disease (PD) affects approximately 10 million individuals worldwide, and its global burden is expected to increase substantially as populations age [1,2]. Neuropathologically, PD is characterised by progressive degeneration of dopaminergic neurons in the substantia nigra pars compacta (SNpc) and by the accumulation of misfolded alpha-synuclein aggregates forming Lewy-type pathology. Although nigrostriatal dopamine depletion accounts for the cardinal motor manifestations of the disease, including bradykinesia, rigidity, and resting tremor, it does not adequately explain the considerable variability in non-motor symptoms, cognitive decline, and rates of disease progression.

Clinical heterogeneity represents one of the major unresolved challenges in PD. Patients with apparently similar clinical presentations at diagnosis may follow markedly different trajectories, ranging from slowly progressive tremor-dominant phenotypes to rapidly evolving disease associated with early cognitive impairment, autonomic dysfunction, hallucinations, postural instability, or REM sleep behaviour disorder. Current clinical and pathological staging systems provide valuable information regarding disease extent but have limited capacity to predict individual disease progression.

### 1.2. Limits of Alpha-Synuclein Propagation Models

The most influential neuropathological model of PD progression remains the Braak staging system, which proposes a stereotypical ascending spread of Lewy pathology from the lower brainstem and olfactory regions to the midbrain and subsequently to limbic and neocortical structures [3]. The concept of alpha-synuclein propagation is further supported by experimental models demonstrating cell-to-cell transmission of pathological alpha-synuclein aggregates, as well as by observations of Lewy-type pathology developing in transplanted foetal dopaminergic neurons in patients with PD [4,5].

However, propagation models alone do not fully explain the selective pattern of neuronal degeneration observed in PD. Neuronal populations exposed to comparable pathological environments may exhibit markedly different susceptibilities. For example, dopaminergic neurons of the ventral tegmental area (VTA) are relatively preserved compared with adjacent SNpc neurons, while calbindin-positive neuronal populations demonstrate increased resistance compared with calbindin-negative ventral-tier SNpc neurons [6]. Furthermore, not all patients follow the classical Braak sequence, highlighting the existence of additional determinants of disease expression and progression [7].

### 1.3. Selective Neuronal Vulnerability as a Biological Explanation for Disease Variability

Increasing evidence suggests that the clinical heterogeneity of PD arises not only from differences in alpha-synuclein distribution but also from intrinsic differences in neuronal biological resilience. The capacity of individual neuronal populations to tolerate pathological alpha-synuclein exposure appears to depend on multiple interacting mechanisms, including calcium homeostasis, mitochondrial and energetic reserve, lysosomal protein clearance, axonal and synaptic architecture, and the inflammatory state of the local cellular environment.

These mechanisms should not be considered as independent pathways but rather as interacting components of the neuronal biological reserve. When multiple vulnerability domains become impaired, neurons may lose the ability to maintain proteostasis, energy production, synaptic integrity, and resistance to inflammatory stress, ultimately resulting in neurodegeneration.

### 1.4. From Mechanisms of Vulnerability to Biological Stratification

Recent advances in biological classification systems, including the SynNeurGe criteria and the Neuronal alpha-Synuclein Disease Integrated Staging System (NSD-ISS), have shifted PD research toward biomarker-based definitions incorporating alpha-synuclein pathology, neurodegeneration, and genetic background [8,9]. The Movement Disorder Society has further emphasised both the opportunities and the challenges associated with implementing biological definitions of PD in research and clinical contexts [10]. Recent validation studies involving cohorts such as PPMI, PASADENA, and SPARK have further supported the potential utility of biologically based staging approaches while highlighting the need for rigorous prospective validation [11].

Despite these important developments, current frameworks primarily define disease presence and stage rather than explaining why some individuals experience a more aggressive neurodegenerative course. A complementary approach may therefore be to identify measurable biological determinants of neuronal susceptibility that could improve prognostic stratification and facilitate mechanism-based clinical trials.

### 1.5. Aim of the Review and Proposed Conceptual Framework

The aim of this review is to critically examine the mechanistic basis of selective neuronal vulnerability in Parkinson’s disease and to explore how these mechanisms may be translated into biomarker-based patient stratification. Specifically, this review integrates evidence from human post-mortem studies, genetic and biomarker research, experimental models, and induced pluripotent stem cell studies to examine five major domains of vulnerability: calcium dysregulation, mitochondrial dysfunction and energetic failure, impaired lysosomal clearance, axonal and synaptic susceptibility, and neuroinflammatory mechanisms.

Based on the integration of these biological domains, we propose the Parkinson’s Vulnerability Index (PVI) as a hypothesis-generating research framework intended to support future biomarker stratification and trial enrichment. Importantly, the PVI should not be considered a validated diagnostic or prognostic tool, and its application in clinical practice requires prospective validation and independent evaluation.

## 2. Materials and Methods

### 2.1. Study Design and Conceptual Framework

This article was designed as a structured critical narrative review aimed at integrating current evidence regarding the mechanisms underlying selective neuronal vulnerability in Parkinson’s disease and evaluating their potential application for biological stratification and precision medicine approaches. A narrative review methodology was deliberately selected because the central objective of this work was not solely to quantify existing evidence, but to integrate multidisciplinary findings derived from human neuropathological studies, genetic investigations, molecular and cellular research, experimental models, biomarker studies, and longitudinal clinical cohorts. Given the heterogeneity of study designs, experimental models, and outcome measures across the included literature, a quantitative meta-analysis would be methodologically inappropriate and potentially misleading. A narrative synthesis is therefore the most suitable approach for addressing the multifactorial nature of selective neuronal vulnerability in PD.

We acknowledge that our structured search strategy and transparent reporting of selection decisions create an expectation of systematic review methodology. However, the hybrid approach involving a structured critical narrative review with systematised search and selection transparency was chosen to balance methodological rigour with the interpretive flexibility required to integrate evidence across multiple biological domains and study types. To meet this expectation, we have enhanced our methodological reporting and provided supplementary materials that align with PRISMA-level transparency to an extent feasible for a narrative review.

A narrative review methodology was deliberately selected because the central objective of this work was not solely to quantify existing evidence, but to integrate multidisciplinary findings derived from human neuropathological studies, genetic investigations, molecular and cellular research, experimental models, biomarker studies, and longitudinal clinical cohorts.

**Figure 1:**
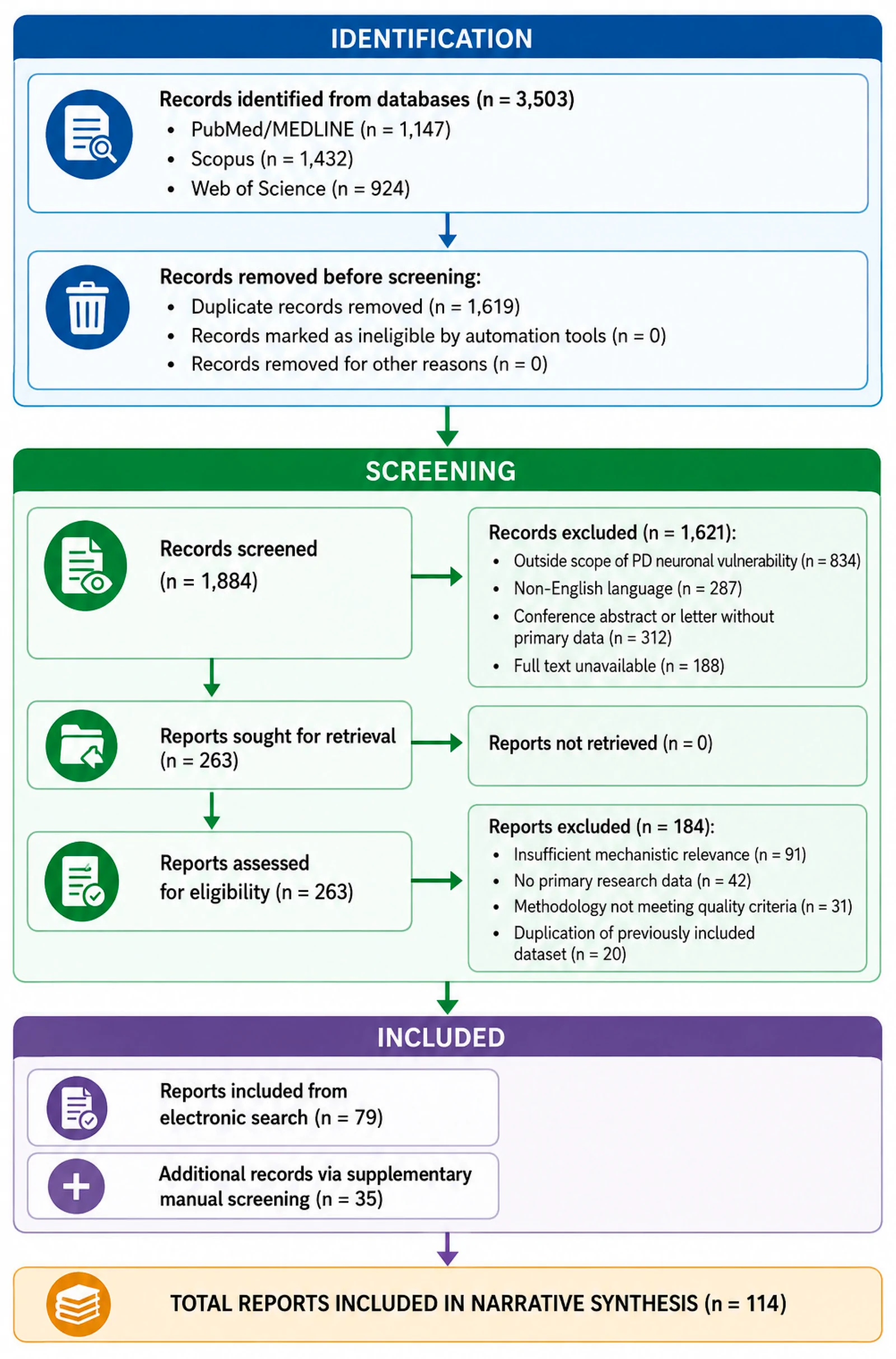
Flow diagram of study identification and selection for the structured critical narrative review. Database searches were conducted in PubMed/MEDLINE, Scopus, and Web of Science from inception to March 2025. Supplementary manual screening of reference lists of key reviews and landmark publications was conducted to capture evidence published after the electronic search cut-off, extending to the date of manuscript submission. The distinction between the primary systematic search and the supplementary manual screening is indicated. SAA = seed amplification assay; PD = Parkinson’s disease. Record counts are reported in Section 2.4. Source: scientific illustrations were generated using BioRender software (BioRender, Toronto, Canada; Version 2026.07.06). The platform was accessed on 6 August 2026 for the creation of this figure.

The review was conducted according to principles of methodological transparency and scientific rigour commonly recommended for high-quality narrative reviews, including a predefined search strategy, explicit eligibility criteria, structured evidence extraction, thematic synthesis, and critical interpretation of the translational relevance of available data. The review was conducted according to the principles of methodological transparency and scientific rigour commonly recommended for high-quality narrative reviews, including a predefined search strategy, explicit eligibility criteria, structured evidence extraction, thematic synthesis, and critical interpretation of the translational relevance of available data. A flow diagram of the complete search and selection strategy is provided as Figure 1.

### 2.2. Literature Sources and Search Strategy

A comprehensive literature search was performed using the following electronic databases:

- **PubMed/MEDLINE;**
- **Scopus;**
- **Web of Science.**

The literature search included articles published from database inception until March 2025.

Only articles published in the English language and available as full-text publications in peer-reviewed journals were considered eligible for inclusion.

The search strategy was developed to identify studies investigating selective neuronal vulnerability, alpha-synuclein pathology, molecular mechanisms of neurodegeneration, biomarkers, disease heterogeneity, and biological stratification in PD. The term “Lewy pathology” was used as a primary search term because it encompasses the full spectrum of alpha-synuclein inclusion pathology including Lewy bodies, Lewy neurites, and pale bodies, consistent with current neuropathological consensus terminology; supplementary searches also used “Lewy body disease” and “Lewy body pathology/pathophysiology” to ensure full coverage. Environmental and demographic modifiers of neuronal vulnerability (pesticide and toxin exposure, traumatic brain injury, age, sex, demographics) were incorporated into the search to reflect the breadth required for the proposed PVI framework. A brief clarification on terminology is warranted.

The term “Lewy body diseases” refers primarily to a defined group of clinical disease entities, principally Parkinson’s disease, dementia with Lewy bodies, and Parkinson’s disease dementia; it carries implicit nosological boundaries that would have narrowed the search in ways inappropriate for the objectives of this review. Similarly, “Lewy body pathophysiology” directs attention toward mechanistic pathways of established disease processes rather than toward the full range of alpha-synuclein inclusion pathology that characterises selective neuronal vulnerability across synucleinopathies.

By contrast, “Lewy pathology” is a purely descriptive, pathology-anchored term that captures the presence of Lewy bodies, Lewy neurites, and pale bodies without presupposing a specific disease entity, disease stage, or clinicopathological correlation. This makes it the most appropriate primary search term for a review whose central question concerns cell-intrinsic determinants of susceptibility to alpha-synuclein inclusion formation across neuronal populations, irrespective of which disease label applies to the affected individual. The supplementary searches using “Lewy body disease” and “Lewy body pathology/pathophysiology” were conducted to capture any relevant evidence that may have been indexed under those alternative terms and not retrieved by the primary search, ensuring that the choice of primary term did not inadvertently exclude relevant literature.

The following filters were applied: English language; full-text availability; peer-reviewed journals; human, animal, or in vitro studies; publication date from database inception to March 2025. No study design filters were applied to ensure comprehensive coverage of the heterogeneous evidence base:

(“Parkinson’s disease” OR “Parkinson disease”) AND (“selective neuronal vulnerability” OR “neuronal susceptibility” OR “neuronal resilience” OR “dopaminergic neuron degeneration” OR “substantia nigra pars compacta”) AND (“alpha-synuclein” OR “Lewy pathology” OR “protein aggregation”) AND (“calcium dysregulation” OR “mitochondrial dysfunction” OR “oxidative stress” OR “lysosomal dysfunction” OR “autophagy impairment” OR “axonal degeneration” OR “synaptic dysfunction” OR “neuroinflammation” OR “microglial activation”) AND (“biomarkers” OR “disease progression” OR “patient stratification” OR “precision medicine”). AND (“environmental factors” OR “environmental exposure” OR “pesticide exposure” OR “toxin exposure” OR “MPTP” OR “rotenone” OR “paraquat” OR “traumatic brain injury” OR “head trauma” OR “age at onset” OR “age” OR “sex” OR “gender” OR “demographics” OR “cognitive reserve” OR “GBA1” OR “glucocerebrosidase” OR “LRRK2” OR “genetic risk factors” OR “disease heterogeneity”)

Additional manual screening of the reference lists of relevant reviews and landmark studies was performed to identify important publications that might not have been captured by the electronic database search. The search strategy incorporates environmental and demographic terms (including pesticide and toxin exposure, traumatic brain injury, age at onset, sex, and cognitive reserve) and genetic risk factor terms (GBA1, glucocerebrosidase, LRRK2) in the AND clause of the search string reported above.

### 2.3. Eligibility Criteria

**Inclusion Criteria**

Studies were eligible for inclusion if they fulfilled one or more of the following criteria:

1. Original peer-reviewed articles investigating mechanisms of selective neuronal vulnerability in PD.
2. Human post-mortem neuropathological studies evaluating regional patterns of alpha-synuclein accumulation, neuronal loss, and differential neuronal susceptibility.
3. Genetic studies evaluating pathogenic mutations, genetic risk factors, and molecular pathways associated with neuronal vulnerability or resilience.
4. Experimental studies using:
  - Animal models of alpha-synuclein pathology;
  - Toxin-based models of dopaminergic degeneration;
  - Cellular models;
  - Induced pluripotent stem cell (iPSC)-derived neuronal systems.
5. Clinical studies evaluating:
  - Disease phenotypes;
  - Progression trajectories;
  - Motor and non-motor heterogeneity;
  - Imaging biomarkers;
  - Fluid biomarkers (CSF, blood, and peripheral tissue markers).
6. Longitudinal cohort studies evaluating predictors of disease progression.
7. High-quality systematic reviews, meta-analyses, and landmark conceptual articles considered essential for understanding the historical development of selective neuronal vulnerability concepts.

**Exclusion Criteria**

Studies were excluded if they met one or more of the following criteria:

- Publications not related to Parkinson’s disease or alpha-synuclein-associated neurodegeneration.
- Studies focusing exclusively on symptomatic treatment without mechanistic implications for neuronal vulnerability.
- Studies not available in English.
- Conference abstracts, meeting proceedings, editorials, letters to the editor, and expert opinions lacking original scientific data.
- Articles without accessible full text.
- Studies with insufficient methodological details preventing adequate interpretation of results.
- Duplicate publications or redundant datasets.
- Studies exclusively involving other neurodegenerative disorders without direct translational relevance to PD.

### 2.4. Study Selection Process

The selection process consisted of multiple sequential stages. Database search (PubMed/MEDLINE, Scopus, Web of Science; electronic search completed: March 2025): total records identified across all three databases = 3503 (PubMed/MEDLINE: n = 1147; Scopus: n = 1432; Web of Science: n = 924). Duplicate removal: duplicates identified and removed using reference management software = 1619; records remaining after deduplication = 1884. Title and abstract screening: records screened = 1884; records excluded = 1621; principal reasons for exclusion: outside the scope of PD neuronal vulnerability, non-English language, conference abstract or letter without primary data, full text unavailable; records taken forward to full-text review = 263. Full-text eligibility assessment: full texts assessed = 263; full texts excluded = 184; principal reasons for exclusion: insufficient mechanistic relevance to selective neuronal vulnerability in PD, no primary research data, methodology not meeting minimum quality criteria, duplication of a previously included dataset; studies included from electronic search = 79. Additional records identified via targeted manual screening of reference lists of key reviews and landmark publications, covering post-March 2025 evidence up to the date of manuscript submission = 35. Total studies included in narrative synthesis = 114, comprising the following distribution by study type: human post-mortem neuropathological studies = 24; genetic and genomic studies = 18; experimental animal or toxin-based models = 15; cellular or iPSC-derived neuronal models = 12; biomarker and neuroimaging studies = 22; longitudinal clinical cohort studies = 15; systematic reviews and meta-analyses included for contextual synthesis = 8. Screeners: title and abstract screening and full-text eligibility assessment were performed independently by two screeners (A. Vamanu, Basic and Clinical Neuroscience, IoPPN, King’s College London, London; A. Ciubotaru, Grigore T. Popa University of Medicine and Pharmacy, Iasi). Disagreement resolution: In cases of disagreement between the two reviewers, consensus was reached by discussion. Unresolved disagreements were adjudicated by the senior author (L. Livinț-Popa). No formal inter-rater reliability statistic was calculated, consistent with the narrative review design. A PRISMA 2020 flow diagram summarising the selection process is recommended and may be required by the editorial team.

First, all records identified through database searches were screened based on their title and abstract. Publications clearly unrelated to PD, neuronal vulnerability, or biomarker stratification were excluded.

Second, the full texts of potentially relevant studies were independently evaluated for scientific relevance, methodological quality, and contribution to the predefined domains of vulnerability.

When multiple publications addressed similar mechanisms, preference was given to studies with:

- Larger sample sizes;
- Longitudinal design;
- Robust experimental methodology;
- Replication across independent cohorts;
- Greater translational relevance.

Seminal historical studies establishing fundamental concepts, such as Braak staging, alpha-synuclein propagation, and major genetic discoveries, were retained regardless of publication date due to their importance in understanding current models of PD pathogenesis. Regarding screening: Title and abstract screening and full-text eligibility assessment were performed independently by two reviewers (A. Vamanu and A. Ciubotaru), as detailed in Section 2.4 above. Formal quality assessment using validated tools (e.g., Newcastle-Ottawa Scale) was not applied; quality was evaluated by assessing replication, sample size adequacy, experimental rigour, and consistency with human neuropathological data. The final distribution of included evidence by study type is reported in Section 2.4 above. Studies were not pooled quantitatively; synthesis was narrative and thematic, consistent with the narrative review design. No formal registration of the review protocol was completed prior to the search; no formal protocol was prospectively registered.

### 2.5. Data Extraction and Evidence Synthesis

Data were systematically extracted from eligible studies according to the following variables:

- Study design;
- Experimental model or patient population;
- Sample characteristics;
- Biological pathway investigated;
- Principal mechanistic findings;
- Associated biomarkers;
- Relationship with neuronal susceptibility or resilience;
- Potential implications for patient stratification.

Because of the expected heterogeneity of the included studies regarding design, methodology, biological models, and outcome measures, a quantitative meta-analysis was not considered appropriate.

Instead, evidence was synthesised using a thematic biological framework, integrating data across complementary research levels:

- Human neuropathology;
- Genetics and genomics;
- Molecular biology;
- Cellular and iPSC models;
- Animal studies;
- Neuroimaging;
- Fluid and tissue biomarkers;
- Longitudinal clinical cohorts.

The strength of evidence was interpreted according to reproducibility across independent studies, consistency between experimental and human data, biological plausibility, and translational potential.

### 2.6. Identification of Major Domains of Selective Neuronal Vulnerability

The five principal domains discussed in this review were selected based on the convergence of evidence from neuropathological, molecular, genetic, and biomarker studies. The selection was guided by three explicit operational criteria: (1) reproducibility across multiple independent biological platforms including human post-mortem tissue, animal models, cellular systems, and clinical biomarker studies; (2) a direct demonstrated link between the mechanism and differential neuronal susceptibility in PD; (3) translational potential, defined as the existence of at least one measurable biomarker or pharmacological target accessible in living patients. No formal statistical tests were performed to select or validate the five domains. No Delphi consensus procedure, inter-rater reliability analysis, quantitative evidence mapping, or systematic scoring of candidate mechanisms against the three criteria was carried out.

The convergence decision was reached through a structured iterative evidence-synthesis process conducted by the primary authorship team (A. Vamanu and A. Ciubotaru), comprising the following sequential steps: First, a long list of candidate biological mechanisms with documented roles in PD pathogenesis was compiled from existing reviews, mechanistic studies, and genetic analyses identified during the literature search. This long list included all mechanisms included in the five domains as well as those classified as excluded or nested (dopamine oxidation, iron and ferroptosis, ER stress, UPS function, vesicular trafficking, DNA damage, neurovascular mechanisms, ageing, sex, epigenetic state, and co-occurring proteinopathies).

Second, each candidate mechanism on the long-list was independently assessed against the three pre-specified criteria. For the reproducibility criterion, the team evaluated whether convergent evidence existed across at least two independent biological platforms (e.g., human post-mortem tissue AND experimental models, or genetics AND biomarker studies). For the differential vulnerability criterion, the team assessed whether the mechanism specifically predicted which cell populations would degenerate compared with those that would be spared, not just whether it contributed to neurodegeneration in general. For the translational potential criterion, the team assessed whether a patient-measurable biomarker or pharmacological target existed that was linked to that mechanism. Third, mechanisms meeting all three criteria were included as primary domains.

Mechanisms meeting fewer than three criteria were classified as either nested within an existing domain (where mechanistic overlap justified this) or excluded with documented justification (detailed in Section 2.6 above). Fourth, the initial domain selection was reviewed and discussed by the full authorship team, with particular attention to whether any mechanism meeting all three criteria had been inadvertently omitted and whether the nested/excluded classifications were justified. This review did not change the five primary domains but refined the justification provided for each exclusion and nesting decision. The authors acknowledge that this process represents a structured expert evidence-synthesis judgement rather than a fully algorithmic or independently validated domain selection procedure. The absence of formal statistical testing or consensus methodology means that the domain selection carries an inherent risk of subjective bias and cannot be considered reproducible in a strict methodological sense.

This is an appropriate limitation of the narrative review design and is consistent with the declared methodology; however, it means that the five domains should be understood as a structured expert proposal for future prospective validation rather than as an empirically derived and externally validated framework. Future iterations of this work should consider applying a formal consensus methodology such as a Delphi process to achieve a more reproducible and externally validated domain selection.

These domains include:

1. Calcium dysregulation and pacemaker-associated metabolic burden;
2. Mitochondrial dysfunction and impaired cellular bioenergetics;
3. Lysosomal dysfunction and defective proteostasis;
4. Axonal architecture, synaptic vulnerability, and impaired neuronal connectivity;
5. Neuroinflammation and glial-mediated mechanisms of neuronal injury.

Although discussed as separate categories, these mechanisms represent highly interconnected biological networks contributing collectively to neuronal resilience or susceptibility. Several additional candidate mechanisms were considered but classified as subsumed within the five domains or as insufficiently characterised for independent stratification. Dopamine oxidation and quinone formation are nested within the mitochondrial domain [11]. Iron accumulation and ferroptosis are considered within the neuroinflammatory framework. Endoplasmic reticulum stress, ubiquitin–proteasome system impairment, and vesicular trafficking defects overlap substantially with the lysosomal domain. DNA damage responses, neurovascular mechanisms, epigenetic state, sex, and co-occurring proteinopathies (amyloid-beta, tau, TDP-43) are important vulnerability modifiers acknowledged in the Discussion section but excluded from the primary PVI domains, because validated stratifying biomarkers in living patients are not yet consistently available.

A detailed justification for the classification of each candidate mechanism is provided below. Dopamine oxidation and quinone formation (nested within the mitochondrial domain). Cytosolic dopamine leaks from storage vesicles and oxidises spontaneously, producing reactive dopamine quinones that covalently modify alpha-synuclein, inhibit the proteasome, and directly impair mitochondrial Complex I function. This mechanism is not independent of the mitochondrial vulnerability domain but rather represents one of the principal upstream drivers of the energetic failure already captured by that domain. Including it as a separate scoring variable would introduce double-counting. No validated patient-level biomarker specifically captures dopamine oxidation status in vivo; neuromelanin-sensitive MRI is an emerging proxy but has not been validated as a vulnerability stratification tool. Iron accumulation and ferroptosis (nested within the neuroinflammatory and oxidative stress domain).

Iron accumulation in the SNpc is a well-documented feature of PD, and elevated iron promotes lipid peroxidation through Fenton chemistry, which can trigger ferroptosis (iron-dependent regulated cell death). This mechanism is mechanistically coupled to the neuroinflammatory environment: activated microglia accumulate iron, which promotes oxidative stress that amplifies microglial activation, and the resulting lipid peroxidation products exacerbate local inflammatory signalling. Neuroimaging markers of iron accumulation (R2*, SWI) are under investigation in PD but have not yet been validated as independent predictors of differential neuronal vulnerability in living patients beyond what existing neuroinflammatory markers capture. Endoplasmic reticulum stress (nested within the lysosomal and proteostatic domain).

ER stress and unfolded protein response activation occur when misfolded proteins accumulate beyond the ER’s handling capacity. In PD, mutant alpha-synuclein and deficient GCase trafficking from ER to Golgi to lysosome are direct causes of ER stress, making this mechanistically downstream of lysosomal dysfunction rather than an independent domain. ER-associated degradation failure also feeds burden onto the autophagy–lysosomal pathway. No validated independent biomarker for ER stress in living PD patients exists at present. The ubiquitin–proteasome system function (nested within the lysosomal and proteostatic domain) and the autophagy–lysosomal pathway (ALP) are compensatory and interconnected degradation systems that share substrate overlap, including alpha-synuclein.

When lysosomal clearance is impaired, proteasomal burden increases, and vice versa. In GBA1-PD, both lysosomal and proteasomal function are impaired as part of the same convergent proteostatic failure. Treating the UPS as a separate scoring domain would require an independent patient-level biomarker, which does not currently exist. Vesicular trafficking (nested within the lysosomal and proteostatic domain). LRRK2 G2019S hyperphosphorylates Rab GTPases that regulate vesicular trafficking to and from lysosomes. GCase misfolding impairs its trafficking from ER to Golgi and onward to lysosomes. Vesicular trafficking defects are therefore mechanistic subcomponents of lysosomal vulnerability that are already partially captured by GCase activity and LRRK2 genotype, and they have no independent validated patient-level biomarker. DNA damage responses (excluded; insufficient evidence for selective neuronal vulnerability).

DNA double-strand breaks, impaired base excision repair, and mitochondrial DNA damage have been reported in PD models and post-mortem tissue, but a reproducible demonstration that DNA damage response status specifically predicts differential vulnerability across neuronal populations in human PD is lacking across the three criteria applied in this review. No validated patient-level biomarker for DDR-based neuronal vulnerability in PD exists. Neurovascular mechanisms are excluded, acting as vulnerability modifiers rather than a cell-intrinsic determinant. Neurovascular coupling, blood–brain barrier integrity, and microvascular function are important context-level modifiers of PD progression.

However, they represent properties of the cellular and vascular microenvironment rather than cell-intrinsic determinants of differential alpha-synuclein susceptibility across defined neuronal populations, which is the criterion applied in domain selection. Ageing (excluded as a scored domain; treated as a universal biological modifier). Ageing is the dominant risk factor for PD and modulates all five selected vulnerability domains (increasing calcium load, reducing mitochondrial reserve, impairing lysosomal clearance, reducing axonal transport fidelity, and amplifying neuroinflammatory tone).

However, ageing is not a cell type-specific vulnerability mechanism but a universal biological context in which the five selected mechanisms operate with increasing impact. Including age as a sixth scoring domain would conflate a non-specific universal modifier with the cell-intrinsic mechanisms the framework aims to capture. Age at onset and disease duration are best handled as covariates in the statistical models used to validate the candidate variable set, not as scored items. Sex is excluded as a scored domain, and is a potential covariate in validation.

Male sex is associated with higher PD incidence, and oestrogen has neuroprotective effects on dopaminergic neurons in experimental models, likely mediated through mitochondrial and anti-inflammatory pathways. However, sex has not been specifically linked to differential neuronal vulnerability across defined cell populations in a manner that produces a measurable patient-level biomarker for stratification. Sex should be included as a covariate and stratification variable in any prospective validation of the candidate variable framework. Epigenetic state (excluded; insufficient translational biomarker evidence). SNCA promoter methylation, histone modifications, and non-coding RNA regulation contribute to alpha-synuclein expression variability and may modulate differential vulnerability. However, reproducible evidence for a specific, cell-type-selective epigenetic vulnerability mechanism that can be measured in living patients with current technology is lacking. This domain is appropriate for future iterations of the framework as epigenetic profiling methods in accessible biological samples mature.

Concomitant proteinopathies (amyloid-beta, tau, TDP-43) are excluded as primary vulnerability domains but are essential as covariates in PVI validation. Co-pathological burden substantially modifies PD clinical course, particularly cognitive trajectory, and validated fluid and imaging biomarkers exist for all three proteins (CSF Aβ42, Aβ42/Aβ40 ratio, CSF p-tau181, p-tau217, amyloid PET, tau PET, and emerging TDP-43 biomarkers). However, these represent extrinsic pathological co-occurrences that modify how existing neuronal vulnerability expresses clinically, rather than cell-intrinsic properties that determine susceptibility to alpha-synuclein pathology specifically. Their exclusion from the core vulnerability domains does not diminish their importance; on the contrary, they must be incorporated as essential covariates in any validation study where cognitive progression is the outcome of interest. Without adjusting for amyloid, tau, TDP-43, APOE genotype, and vascular burden, the independent contribution of vulnerability markers to cognitive outcomes cannot be properly evaluated. This is not merely a methodological nicety but a fundamental requirement for valid inference: failure to include these covariates would risk attributing to vulnerability markers an effect that is actually driven by co-pathology. Future model development should therefore test whether adding co-pathological biomarkers improves prediction of cognitive outcomes beyond the five primary vulnerability domains alone, and whether the vulnerability markers remain significant predictors after covariate adjustment. Section 5.2 provides a detailed framework for the inclusion of these covariates in validation studies.

### 2.7. Development of the Parkinson’s Vulnerability Index (PVI)

The Parkinson’s Vulnerability Index (PVI) was developed as a conceptual research model derived from the integration of the identified vulnerability domains. The development of the PVI was motivated by a specific and clearly identifiable clinical and scientific gap. Parkinson’s disease is currently classified using clinical criteria and, more recently, biological staging systems (SynNeurGe, NSD-ISS) that define disease presence and stage based on the distribution of alpha-synuclein pathology, dopaminergic neurodegeneration, and associated genetic markers. These systems are valuable for determining whether a patient has PD and how far it has progressed structurally and biologically.

However, they do not directly address a question of growing clinical importance: among patients who already meet criteria for PD at diagnosis, why do some develop dementia within five years while others remain cognitively intact for two decades? Why do some develop a severe non-motor phenotype while others follow a predominantly tremor-dominant, slowly progressive course? These differences in clinical trajectory within the PD spectrum cannot be adequately explained by alpha-synuclein spread models alone and are not predicted by current staging systems at the individual patient level.

The central hypothesis driving the PVI design is that these individual differences in clinical trajectory reflect differences in the biological vulnerability of specific neuronal populations: their capacity to resist alpha-synuclein pathology given their genetic make-up, lysosomal function, mitochondrial reserve, axonal architecture, and local inflammatory microenvironment. If this hypothesis is correct, measuring biological vulnerability at or near the time of diagnosis should provide prognostic information that is complementary to, and partially independent of, existing staging markers.

A second motivating observation was the consistent failure of neuroprotective clinical trials in PD to show disease-modifying effects in broad, clinically defined populations. Trials enrolling patients by diagnosis alone, without pre-specifying which biological mechanism was most active in each participant, enrol mechanistically heterogeneous populations that dilute the therapeutic signal of mechanism-targeted interventions to the point of non-detectability.

A vulnerability stratification tool could enable enriched enrolment of the subgroups most likely to respond to each mechanism-targeted agent. These two motivating observations shaped the key design choices of the PVI. The decision to combine genetic, enzymatic, protein-seeding, imaging, olfactory, and cognitive measures into a single framework reflects the multi-domain nature of neuronal vulnerability: no single biomarker captures the full biological risk profile at diagnosis. The decision to present the PVI as a hypothesis-generating research framework rather than as a validated clinical tool reflects the current evidence state: a strong biological rationale exists for each component, but the combination has never been prospectively tested as a composite predictor and no empirical optimisation of weights or thresholds has been performed. The decision to position the PVI as complementary to SynNeurGe and NSD-ISS reflects the view that vulnerability biology operates within, not instead of, the established alpha-synuclein and neurodegeneration framework.

The intended translational pathway is sequential: (1) prospective validation of the composite PVI score in existing cohort studies (e.g., PPMI, Oxford Discovery) with pre-specified clinical endpoints; (2) if validated, use of PVI-stratified enrolment in mechanism-targeted clinical trials to test whether enriched populations show larger treatment effects than heterogeneous ones; (3) only if steps (1) and (2) provide converging positive evidence, with consideration of vulnerability stratification within clinical guidelines, as well as the full ethical and counselling infrastructure the framework requires. The PVI is at step (1) of this pathway and must not be used clinically outside a validation research protocol.

The proposed framework aims to organise biological determinants of neuronal susceptibility into a multidimensional model incorporating molecular, cellular, genetic, imaging, and fluid biomarker information.

Following the reviewer’s recommendation, we have restructured the proposed PVI framework into four separate modules based on the temporal and biological nature of the variables. This restructuring addresses the temporal inconsistency identified in the original proposal, where baseline vulnerability markers were combined with longitudinal outcome variables in a single composite index.

**Module 1: Vulnerability Biomarkers (Cell-Intrinsic Properties).** These markers reflect biological properties that exist before pathology is established and influence the capacity of neurons to tolerate alpha-synuclein exposure. They are the primary targets for enrichment in mechanism-targeted clinical trials. Included variables: GBA1 genotype, LRRK2 G2019S genotype, CSF GCase activity, dried blood spot GCase activity.

**Module 2: Pathology and Staging Biomarkers (Active Disease Processes).** These markers reflect the current state of alpha-synuclein pathology and established neurodegeneration. They provide biological staging information that is complementary to vulnerability markers. Included variables: CSF alpha-synuclein SAA (pathology marker), DAT-SPECT striatal binding ratio (neurodegeneration marker).

**Module 3: Baseline Clinical Measures (Current Functional Status).** These markers reflect the patient’s functional status at the time of assessment. They are disease-state markers that correlate with existing neurodegeneration but do not measure intrinsic vulnerability. Included variables: MoCA score at diagnosis, UPSIT olfactory function.

**Module 4: Prospective Outcome Measures (Longitudinal Change).** These variables represent changes that occur over time and cannot be scored at baseline. They are the outcomes to be predicted rather than components of a predictive index. Included variables: 12-month MoCA decline (≥2 points), informant-corroborated cognitive concern emerging during follow-up.

**Important:** The PVI score should be calculated using only Modules 1, 2, and 3 at baseline. Module 4 variables are not components of the baseline PVI score; they are outcome measures to be used in prospective validation studies. The illustrative point weights presented in this review apply only to Modules 1–3 and are hypothesis-generating values requiring empirical optimisation in prospective cohort data. The combined baseline score (Modules 1–3) should be tested for its ability to predict Module 4 outcomes, with the understanding that the relationship between baseline vulnerability and longitudinal outcomes is the hypothesis to be tested, not a pre-existing assumption. This modular structure allows separate evaluation of whether vulnerability markers (Module 1), pathology markers (Module 2), or clinical state markers (Module 3) drive predictive performance, addressing the circularity concern raised in the peer review process.

The assessment of each candidate mechanism against the three pre-specified criteria is documented in Section 2.6, and the final classification decisions are justified there. The modular structure of the PVI is designed to be complementary to existing staging systems such as SynNeurGe and NSD-ISS, not to replace them. A patient could be classified biologically by SynNeurGe or staged by NSD-ISS, then further stratified within a research cohort by a vulnerability score based on Modules 1–3. Whether this additional layer improves prediction beyond existing biological staging remains an empirical question and is one of the central validation tasks proposed by this review.

Second, the proposed point weights reflect qualitative biological plausibility and evidence maturity; they are not statistically derived regression coefficients and should not be interpreted as empirically validated risk estimates. The weights are illustrative candidate values intended to organise the variables into a testable research framework. Any future validated vulnerability model must derive weights from prospective cohort data using appropriate statistical methods (e.g., multivariable regression, survival analysis, or machine learning) with internal and external validation. The relative weighting (e.g., 3 points for high-risk GBA1 variants versus 1 point for SAA positivity) reflects the current relative maturity of the evidence base for each marker, not an empirically derived contribution to risk prediction. Third, biomarker thresholds are drawn from individual source studies and require local recalibration—they are provisional research anchors, not universal clinical cut-offs. The point values and thresholds presented in this review must not be used for individual patient management, genetic disclosure, or treatment decisions outside a research protocol until prospective validation has been completed.

An important caveat regarding biomarker thresholds must be emphasised: the biomarker measurements described in this review cannot be combined into a universal scoring system yet. The thresholds cited for GCase activity, alpha-synuclein SAA, DAT-SPECT, MoCA, and UPSIT are derived from individual source studies using specific assay platforms, patient populations, and laboratory conditions. These thresholds are not interchangeable across laboratories, assay platforms, or patient populations without local recalibration and independent validation. The proposed point weights are illustrative and not derived from a unified dataset. Consequently, the PVI is presented as a conceptual framework for organising candidate biomarkers rather than a standardised scoring instrument. Any future attempt to create a universal scoring system must address assay standardisation, cross-platform comparability, and population-specific reference ranges before the PVI can be meaningfully applied across different clinical and research settings. The primary contribution of this review is therefore to identify which biomarkers should be measured and how they might be combined, not to provide a validated algorithm for their combination.

### 2.8. Quality Assessment and Methodological Considerations

Given the narrative nature of this review, a formal risk-of-bias assessment tool used in systematic reviews (e.g., Cochrane Risk of Bias or Newcastle–Ottawa Scale) was not applied.

However, studies were critically evaluated based on:

- Methodological robustness;
- Adequacy of experimental design;
- Sample size;
- Replication of findings;
- Consistency with human pathological data;
- Translational relevance.

Greater weight was assigned to convergent evidence reproduced across multiple methodological platforms, including human tissue studies, genetic analyses, biomarker studies, and experimental models.

### 2.9. Methodological Limitations

The authors acknowledge that the narrative review design carries an inherent risk of selection bias and does not provide quantitative effect estimates.

Additionally, heterogeneity among experimental models, patient populations, biomarker assays, and clinical endpoints limits direct comparison between studies.

Nevertheless, this integrative methodology is particularly suitable for addressing the multifactorial nature of selective neuronal vulnerability in PD, allowing the development of a biologically oriented framework that bridges basic science discoveries with future precision medicine applications.

**Figure 2:**
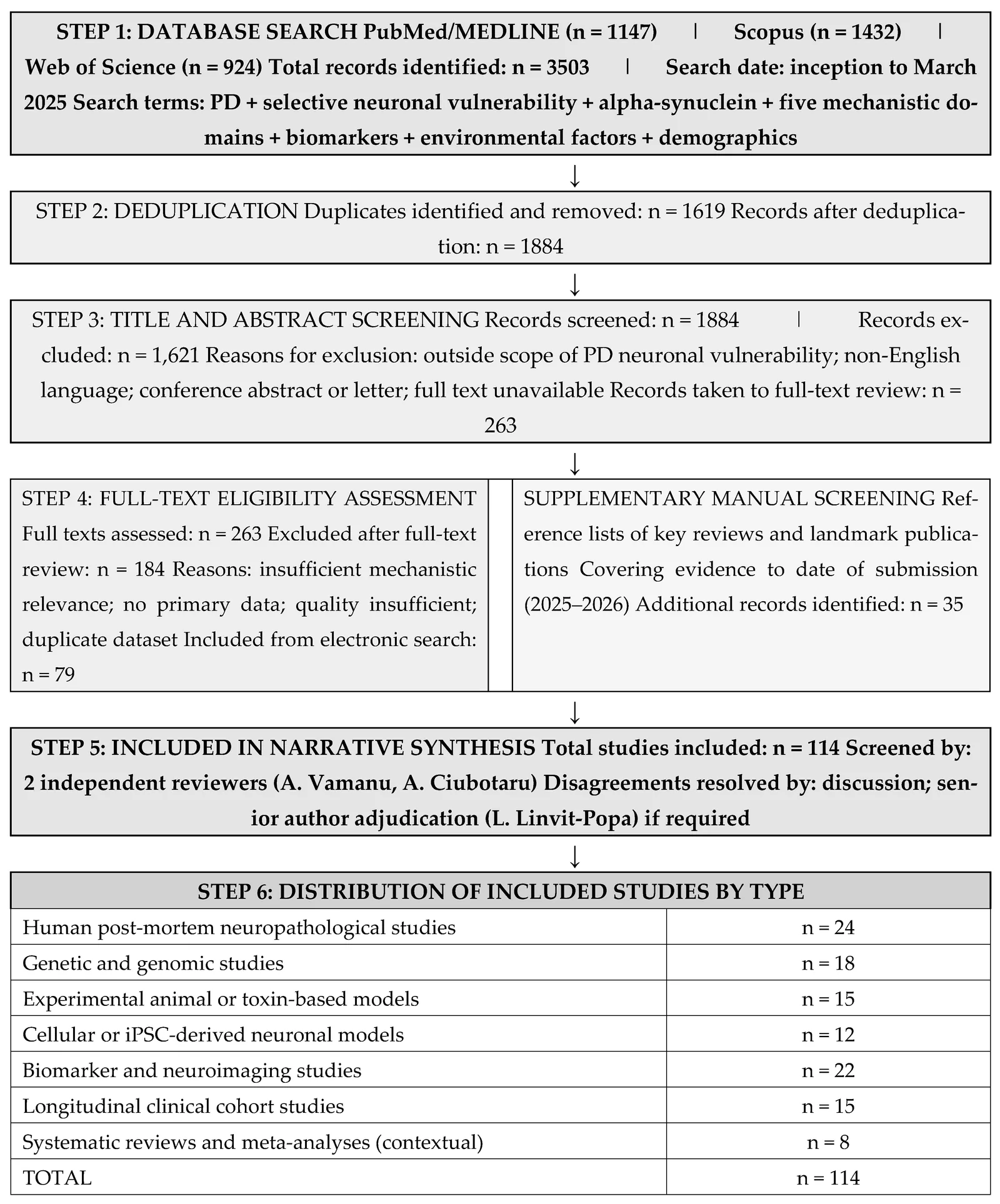
Decision-tree (algorithmic) representation of the literature search and selection strategy for this structured critical narrative review. Database search was conducted in PubMed/MEDLINE, Scopus, and Web of Science from inception to March 2025; supplementary manual screening extended to date of manuscript submission. Record counts are reported in Section 2.4. SAA = seed amplification assay; iPSC = induced pluripotent stem cell; PD = Parkinson’s disease.

To mitigate the risk of selection bias and enhance transparency, we have provided a comprehensive evidence table listing all included studies with their design, population, vulnerability domain, principal findings, limitations, and evidence level. Additionally, we have documented all exclusion decisions with explicit counts and reasons in the flow diagram (Figure 2). These measures allow readers to evaluate the evidentiary basis for our conclusions and the comparative judgments regarding translational maturity of each vulnerability domain.

## 3. Results: Five Biological Mechanisms of Selective Neuronal Vulnerability

The five mechanisms described below each contribute to whether a neuron can tolerate alpha-synuclein exposure. They should be understood as interacting forms of biological reserve rather than as isolated pathways. In vulnerable neurons, calcium stress, energetic burden, impaired lysosomal clearance, long unmyelinated axonal architecture and inflammatory activation place their greatest functional burden on distal axonal and synaptic compartments in neurons with long projection fields. These five mechanisms represent fundamental cellular processes relevant across diverse cell types, including non-neuronal cells and in vitro models without synaptic architecture; their clinical relevance in PD reflects the exceptional metabolic demands of specific neuronal populations.

Early functional failure is most likely to become clinically detectable. These five mechanisms (calcium homeostasis, mitochondrial bioenergetics, lysosomal protein clearance, vesicular and axonal transport, and inflammatory signalling) are fundamental cellular processes that operate across diverse cell types, including non-neuronal cells and in vitro systems that do not form synapses. Their demonstrated relevance in such models does not diminish their importance in PD neurobiology.

The argument for selective neuronal vulnerability rests on a different premise: the QUANTITATIVE BURDEN imposed on these housekeeping processes varies enormously between cell types and that specific neuronal populations, particularly SNpc dopaminergic neurons, impose an exceptional quantitative demand on each of these systems simultaneously. In non-neuronal cells and in most other neuronal populations, calcium influx is modest and tightly regulated; in SNpc neurons, it is driven by lifelong autonomous pacemaking through L-type channels at a rate and amplitude that produces disproportionate mitochondrial calcium loading.

Mitochondrial function must support energy demands across >150,000 unmyelinated axonal terminals per neuron; in dividing cells or in shorter-projecting neurons, this demand is orders of magnitude lower. Lysosomal clearance must handle high endogenous alpha-synuclein concentrations under conditions of dopamine-mediated lysosomal impairment that are specific to dopaminergic cells. The neuroinflammatory microenvironment of the SNpc, with its exceptionally high microglial density and heightened baseline activation, amplifies inflammatory stress to a degree not present in most other brain regions or in vitro systems. This distinction between mechanism universality and quantitative burden also explains why in vitro data showing these mechanisms operating in non-synaptic cells are relevant to, but do not refute, the vulnerability argument. When GBA1-deficient iPSC-derived dopaminergic neurons show greater susceptibility to alpha-synuclein PFF seeding than cortical neurons from the same donor under identical conditions, this demonstrates a cell type-intrinsic vulnerability that operates independently of synaptic architecture.

The vulnerability is real and measurable; what differs between cell types is the quantitative context in which the same housekeeping machinery must operate, not the machinery itself. The synaptic terminal is identified as the locus of early clinical consequences in PD neurons specifically, not because the mechanisms only operate there, but because it is the most distal and most energetically demanding compartment of the neuronal populations most affected by PD, and therefore the site where cumulative housekeeping failure first becomes functionally detectable (see Figure 2 and Table 1).

### 3.1. Calcium Overload

Nerve cells in the SNpc need to fire continuously throughout life to keep dopamine levels stable in the brain. As people age, these cells increasingly rely on L-type calcium channels to support autonomous pacemaking, which increases calcium entry and places sustained metabolic stress on mitochondria [12,13]. Excess intracellular calcium contributes to mitochondrial oxidative stress and may facilitate alpha-synuclein aggregation, creating a convergence between excitability, energy demand and protein handling.

Nerve cells that produce the calcium-buffering protein calbindin D28k can partially buffer this calcium load and are therefore relatively protected, which helps explain the pattern of selective cell loss seen in post-mortem brain studies [6]. A further calcium burden may come from T-type calcium channels enriched in vulnerable SNpc neurons [14]. The STEADY-PD III trial tested whether blocking calcium channels with isradipine could slow PD progression, but did not demonstrate clinical benefit [15]. This result weakens the immediate therapeutic argument for isradipine but does not remove calcium stress from the biology of selective vulnerability.

### 3.2. Energy Failure in Long, Uninsulated Nerve Fibres

A single dopamine neuron in the human SNpc extends fibres that connect to over 150,000 target sites across the brain [16]. These fibres are largely unmyelinated and therefore lack the myelin sheath that insulates most long-range axons; maintaining electrical signalling across their full extent consequently imposes a disproportionate energy demand. Each connection point also requires local mitochondrial support, and those mitochondria must be transported from the cell body to the distal axon and synaptic terminal.

In PD, the main energy-producing complex inside mitochondria, Complex I, is significantly impaired, and the deficit is particularly evident in the SNpc rather than regions such as the cerebellum [17]. Alpha-synuclein can worsen this problem by accumulating in mitochondria and impairing Complex I function directly [18]. Work on dopaminergic neuron architecture has shown that axonal arborisation size and metabolic burden are major contributors to vulnerability [19]. Dopamine itself adds another hazard: when cytosolic dopamine is oxidised, it produces reactive dopamine quinones that can disrupt mitochondrial and lysosomal function [20]. Contemporary reviews of alpha-synuclein biology and neuronal vulnerability increasingly place mitochondrial stress, lysosomal dysfunction, calcium imbalance and synaptic failure into a shared pathogenic framework [21,22].

### 3.3. Lysosomal Failure and the GBA1 Connection

Under physiological and pathological conditions, misfolded or damaged proteins, including alpha-synuclein, accumulate over time. Whether alpha-synuclein is continuously produced in misfolded form under normal conditions remains under investigation; current evidence indicates that misfolding and aggregation are promoted by specific genetic, metabolic, and environmental stressors rather than being constitutive processes. Under normal conditions, lysosomes and autophagy pathways break these proteins down and recycle their components. Alpha-synuclein is partly handled through chaperone-mediated autophagy, and impaired degradation of mutant or aggregated alpha-synuclein can promote accumulation and toxicity [23].

The most common genetic risk factor linked to lysosomal vulnerability in PD is variation in the GBA1 gene, which encodes glucocerebrosidase (GCase). GBA1 mutations are among the most common genetic risk factors for PD [24]. When GCase function is reduced, glucosylceramide and related lipids accumulate, lysosomal handling becomes less efficient, and alpha-synuclein aggregation is favoured. Accumulated alpha-synuclein can then further inhibit GCase trafficking and function, generating a bidirectional pathogenic loop that has been demonstrated in experimental and human cellular models [25,26,27]. Recent reviews have extended this pathway by linking GBA1-PD to lipid metabolism, autophagy, endoplasmic reticulum stress, mitochondrial function, and therapeutic targeting [27]. The clinical implications of GBA1 variant complexity (variant classification, penetrance variability, ancestry-related differences, and the scope of the PVI framework as applied to GBA1 carriers) are discussed in full in Section 4. Variant classification, zygosity, ancestry, penetrance, and scoring scope are addressed in Section 4 of this review. The concept of variable penetrance deserves explicit discussion because it is frequently misunderstood in the context of GBA1 and PD risk. Penetrance in this context refers to the proportion of individuals carrying a specific GBA1 variant who develop PD during their lifetime. For the N370S variant in heterozygous form (the most common GBA1 variant associated with PD in Ashkenazi Jewish populations), the lifetime risk of PD is estimated at approximately 10 to 30 percent, meaning that the large majority of N370S heterozygotes will not develop PD [28,29,30,31].

For more severe variants such as L444P in heterozygous form, the lifetime risk estimates are higher but still substantially below 100 percent, and the risk differs between ancestral populations because the same variant may co-occur with different genetic modifiers depending on population background [32]. Recombinant alleles (RecNciI, RecA) arise from recombination events between GBA1 and its pseudogene GBAP1 and result in multiple simultaneous amino acid changes; they are associated with severe Gaucher disease when biallelic and with elevated PD risk when heterozygous, but their PD penetrance remains incompletely characterised because of their rarity and because they are frequently misidentified on standard sequencing panels that do not include pseudogene-aware analysis.

Complex alleles (e.g., the L444P allele that also carries E326K on the same chromosome) can produce different enzyme activity and clinical outcomes than what the individual component variants would predict. Some variants commonly listed in GBA1 risk analyses (including E326K and T369M) are more accurately described as risk modifiers rather than pathogenic variants, conferring a modest increase in PD risk that does not clearly rise to the level of penetrant pathogenicity and should not be scored equivalently to confirmed pathogenic variants in a research stratification framework. For all these reasons, the PVI framework must specify that the points assigned for GBA1 variants apply only to confirmed heterozygous carriers of pathogenic GBA1 variants as defined by current classification criteria (ClinVar pathogenic or likely pathogenic); variants of uncertain significance should not contribute to the score until reclassified; and biallelic GBA1 mutations causing overt Gaucher disease represent a distinct clinical and genetic entity that is outside the scope of this framework.

The clinical consequence of GBA1 variation is measurable but heterogeneous. Across cohort studies, GBA1 carriers tend to develop cognitive impairment earlier than non-carriers, show a heavier non-motor burden, and have more widespread Lewy pathology [28,29]. LRRK2 G2019S disrupts lysosomal biology through a different mechanism involving Rab phosphorylation, and combined GBA1 and LRRK2 biology may further modify disease phenotype [30]. More recent work has treated GBA1-PD as a distinct clinical and mechanistic entity and has refined the importance of variant severity [31,32]. Longitudinal and meta-analytic studies further suggest that sex, cognitive reserve, and broader molecular context modify risk [33,34,35,36,37].

### 3.4. Connection Architecture and the Vulnerability of Synaptic Tips

The way a nerve cell is structured influences vulnerability independently of its molecular properties. As discussed in Section 3.2, SNpc dopamine neurons maintain extremely extensive axonal and synaptic networks. When axonal transport begins to fail, which can happen early in PD, the distal terminals of these long projections are likely to be starved first of energy and waste-clearance capacity [38]. Post-mortem and clinicopathological studies also suggest that synaptic dysfunction and synaptic loss are often more closely related to cognitive impairment than the mere presence of Lewy bodies [39].

Importantly, even when alpha-synuclein seeds reach a neuron via a connected pathway, damage is not uniform across all cell types. Within the pedunculopontine nucleus, for example, cholinergic neurons develop pathology while neighbouring non-cholinergic neurons receiving related inputs may be relatively spared [40]. Recent single-cell and single-nucleus profiling of human substantia nigra has strengthened this point by showing that PD vulnerability is shaped by molecularly distinct neuronal and glial states, rather than by anatomical exposure alone [41,42].

### 3.5. Neuroinflammation: The Brain’s Immune Response

The SNpc contains a dense microglial population, and extracellular alpha-synuclein released from stressed or dying neurons can activate microglia through innate immune pathways [43]. Recent evidence supports a complex interaction between alpha-synuclein species and microglial activation, with microglia potentially contributing both to clearance and to inflammatory amplification [44]. Single-nucleus transcriptomic analysis of the human midbrain has identified glial activation signatures and PD-associated neuronal transcriptional states, supporting the view that neuroinflammation is an integral component of the local vulnerability environment rather than a purely secondary reaction to neuronal death [45].

**Figure 3:**
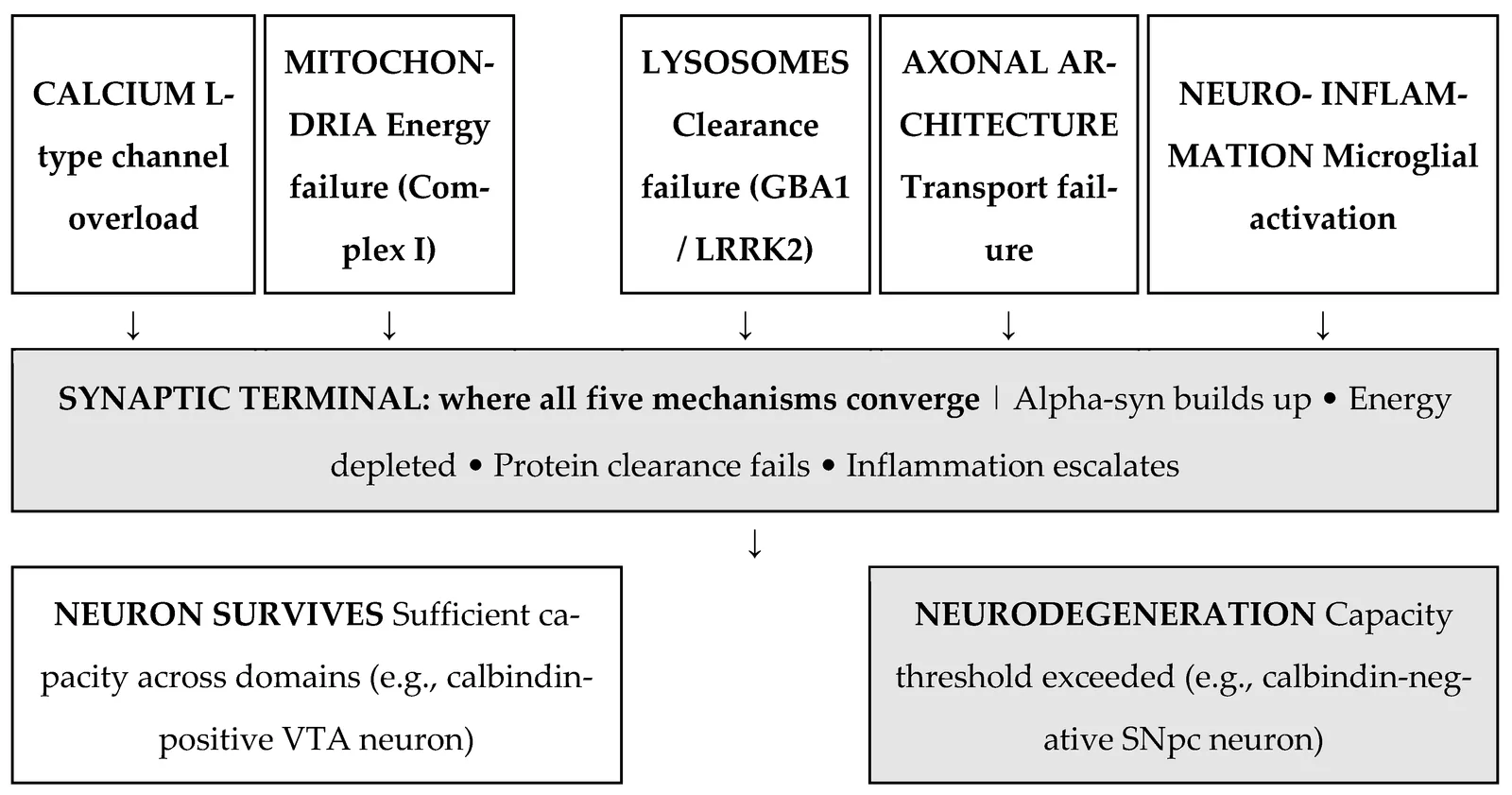
How the five vulnerability mechanisms converge at the nerve cell’s synaptic terminal. All five mechanisms ultimately impair the synapse, the connection point between nerve cells. When multiple mechanisms fail together, the neuron crosses the threshold from stressed to degenerating. Neurons with sufficient reserve across all five domains (such as calbindin-positive VTA neurons) survive; those without it (such as calbindin-negative SNpc ventral-tier neurons) are lost. SNpc = substantia nigra pars compacta; VTA = ventral tegmental area; alpha-synuclein.

Activated microglia can drive astrocytes into neurotoxic reactive states, amplifying local oxidative and inflammatory stress [46]. Recent experimental work on complement C4 and astrocyte-mediated neuroinflammation further supports the idea that astrocytes can contribute directly to synaptic injury and alpha-synuclein pathology under inflammatory conditions [47]. This self-reinforcing glial cycle may help explain why, once SNpc degeneration begins, it can become progressively harder for vulnerable neurons to maintain biological reserve (Figure 3, Table 1 and Table 2).

## 4. The GBA1 Pathway: From Biology to the Clinic

### 4.1. GBA1-PD as a Clinically and Mechanistically Informative Subgroup Within the PD Spectrum

Of all the biological mechanisms described above, the lysosomal pathway is the most immediately actionable from a translational perspective, and GBA1 is its clearest clinical entry point. This evidence justifies systematic consideration of the GBA1 pathway in research stratification and trial enrichment design. Whether universal genetic screening can be implemented in routine practice without agreed counselling, disclosure, and follow-up pathways remains unclear.

Compared with matched PD patients who do not carry a GBA1 mutation, GBA1 carriers generally show earlier cognitive decline, more severe non-motor symptoms, and greater risk of dementia, although the magnitude of risk varies according to variant type and cohort characteristics [28,29,35]. Their post-mortem brains more often show widespread Lewy pathology, including cortical involvement in regions relevant to memory, attention and executive function. When lysosomal dysfunction is combined with other genetic or biological stressors, the clinical phenotype may become still more complex [30].

This dose–response relationship between lysosomal impairment and clinical severity is the clearest existing demonstration in PD research that a specific biological vulnerability mechanism produces a measurable, probabilistic, group-level clinical signal, one that is reproducible across cohort studies but does not permit individual outcome prediction. It is important to emphasise, however, that GBA1-PD should be understood as a clinically and mechanistically informative subgroup within the broader PD spectrum rather than a categorically distinct disease entity. There is substantial biological and clinical overlap with idiopathic PD, individual outcomes are not reliably predictable from GBA1 genotype alone, and the magnitude of risk varies considerably with variant severity, ancestry, zygosity, and additional genetic and environmental context.

In summary, GBA1-PD is best understood as a clinically and mechanistically informative subgroup within the continuous PD spectrum. It does not constitute a separate disease entity with a distinct nosological boundary. The overlap with idiopathic PD is substantial at the pathological, biomarker, and clinical levels, and clinicians should not communicate a GBA1-positive result as conferring a categorically different diagnosis or a deterministic clinical trajectory. The appropriate framing is one of modified probabilistic risk within the PD spectrum, not disease reclassification.

### 4.2. Measuring GCase in Clinical Practice

GCase enzymatic activity can be measured practically in two ways. A dried blood spot test measures GCase activity from a small blood sample collected on a card, while cerebrospinal fluid (CSF) measurement requires lumbar puncture. Published thresholds, including dried blood spot GCase activity below 2.8 nmol/h/mg protein and CSF GCase activity below approximately 39 nmol/h/mL, have been associated with elevated risk or heavier Lewy pathology in source studies [48,49]. These values should be interpreted as research thresholds rather than universal clinical cut-offs. GCase activity is substantially affected by pre-analytical variables including sample type, substrate concentration and specificity, buffer composition, pH, incubation time, blood cell composition, storage conditions, freeze–thaw cycles, and laboratory-specific calibration; reference ranges from individual source studies cannot be generalised across laboratories without local recalibration.

Critically, peripheral GCase measurements require explicit interpretation with respect to their limitations. A fundamental limitation that must be emphasised is that peripheral blood GCase activity, whether measured in whole blood, plasma, leucocytes, or dried blood spots, does not necessarily reflect GCase function in brain neurons or central nervous system lysosomes.

Erythrocyte GCase is predominantly the lysosomal isoform but is expressed at high concentrations in red blood cells, which may dominate the whole-blood signal and obscure the cellular signal of interest. Plasma GCase reflects secreted enzyme rather than intracellular lysosomal activity. Leucocyte GCase activity provides a closer cellular analogue to neuronal lysosomes but is technically demanding, requires specialised laboratory processing, and is rarely used in routine research practice. None of these peripheral measures has been directly validated against neuronal GCase activity in human post-mortem brain tissue or against clinical outcomes in independent PD cohorts using the same assay platform. Furthermore, the relationship between peripheral GCase reduction and brain GCase reduction may be influenced by systemic factors including inflammation, metabolic state, and blood cell composition that are unrelated to neuronal lysosomal function. Peripheral GCase activity should therefore be understood as an accessible surrogate biomarker with biological plausibility rather than a direct window into the central lysosomal environment.

The implications of this limitation for the proposed PVI framework are substantial. The GCase thresholds cited in Table 2 of this review (DBS: <2.8 nmol/h/mg from L represent reference anchors derived from two specific studies using specific assay conditions, and they should be used only as starting points for prospective calibration within locally established laboratory settings. Any research group wishing to use GCase activity as part of a vulnerability stratification framework must first establish their own platform-specific reference range in an appropriate comparison population before applying any threshold to patient data. The PVI framework must therefore specify that GCase scoring applies only within laboratory settings that have established local reference ranges using the same assay conditions as the source studies, and that peripheral GCase values should not be interpreted as direct measures of brain lysosomal function.

GCase activity is substantially affected by pre-analytical variables including sample type, substrate concentration and specificity, buffer composition, pH, incubation time, blood cell composition, storage conditions, freeze–thaw cycles, and laboratory-specific calibration; reference ranges from individual source studies cannot be generalised across laboratories without local recalibration.

Recommendations for GCase measurement in PVI validation studies: Given the limitations of peripheral GCase measurements, we recommend the following principles for any validation study incorporating GCase activity into the PVI framework: (1) GCase activity should be measured using a standardised, validated assay protocol with documented pre-analytical procedures; (2) laboratory-specific reference ranges should be established in an appropriate comparison population before applying any threshold to patient data; (3) the relationship between peripheral GCase activity and clinical outcomes should be examined separately for different sample types (DBS, plasma, leucocytes) to determine which provides the strongest signal; (4) wherever feasible, CSF GCase measurement should be used as the reference standard against which peripheral measures are validated; (5) when peripheral measures are used, the results should be interpreted cautiously and the limitations explicitly acknowledged in any reporting; (6) the predictive value of GCase activity should be evaluated in models that include other covariates (amyloid, tau, APOE, vascular burden) to determine whether GCase adds independent predictive value beyond established modifiers. These principles are intended to guide methodologically rigorous validation studies and to prevent overinterpretation of peripheral GCase measurements in clinical or research contexts.

Blood-based alpha-synuclein SAA assays using extracellular vesicle-enriched or phosphorylated substrates are methodologically distinct from validated CSF-based assays and results cannot be assumed equivalent without independent comparative validation. A further important limitation is that peripheral blood GCase activity, whether measured in whole blood, plasma, leucocytes, or dried blood spots, is not necessarily a direct or proportionate measure of GCase function in brain neurons or central nervous system lysosomes. Erythrocyte GCase is predominantly the lysosomal isoform but is expressed at high concentrations in red blood cells, which may dominate the whole-blood signal. Plasma GCase reflects a secreted enzyme rather than intracellular lysosomal activity. Leucocyte GCase activity provides a closer cellular analogue to neuronal lysosomes but is technically demanding and rarely used in routine research practice.

None of these peripheral measures has been directly validated against neuronal GCase activity in human post-mortem brain tissue or against clinical outcomes in independent PD cohorts using the same assay platform. Peripheral GCase activity should therefore be understood as an accessible surrogate biomarker with biological plausibility rather than a direct window into the central lysosomal environment. In practical terms, this means that the GCase thresholds cited in Table 2 of this review (DBS: <2.8 nmol/h/mg; CSF: <39 nmol/h/mL) represent reference anchors derived from two specific studies using specific assay conditions, and they should be used only as starting points for prospective calibration within locally established laboratory settings. Any research group wishing to use GCase activity as part of a vulnerability stratification framework must first establish their own platform-specific reference range in an appropriate comparison population before applying any threshold to patient data.

The same principle applies to alpha-synuclein SAA: the diagnostic performance characteristics reported in the PPMI cohort and confirmatory studies were obtained using specific CSF-based assay protocols, and these must not be assumed to apply to blood-based, plasma-based, or EV-derived assay variants, which represent distinct and as yet incompletely validated biomarker platforms.

An important finding is that GCase activity is also reduced in a proportion of PD patients who do not carry a GBA1 mutation [50]. This may reflect downstream inhibition of lysosomal function by alpha-synuclein itself. As a result, low GCase activity may identify a broader at-risk biological subgroup than genetic testing alone, although this requires prospective validation before it can be used for individual-level prognostication.

### 4.3. The Alpha-Synuclein Seeding Test (SAA)

In the large PPMI cohort, CSF alpha-synuclein SAA identified clinically diagnosed PD with high sensitivity and specificity and revealed biological heterogeneity within genetic and clinical subgroups [51]. Earlier comparative work also supported the diagnostic potential of CSF alpha-synuclein seed amplification [52]. More recent studies have refined the interpretation of SAA by examining amplification kinetics, prodromal conversion risk, negative SAA results in clinically diagnosed PD, and the relationship between CSF assays and tissue-based alpha-synuclein testing [53,54,55,56].

The combination of genetic status, GCase activity and SAA status is likely to be more informative than any single measure. A GBA1 carrier with reduced GCase activity and positive SAA may plausibly have higher biological vulnerability than a non-carrier with preserved GCase activity, but this needs prospective testing before it can be used for prognosis or management.

## 5. The Parkinson’s Vulnerability Index (PVI): A Research Stratification Proposal

The five vulnerability mechanisms, the genetic evidence, and the available biomarker data provide a rationale for proposing a structured research score: the Parkinson’s Vulnerability Index, or PVI. The purpose of the PVI is to organise biological risk information in a way that can be tested prospectively. It is intended to support cohort stratification, trial enrichment and hypothesis generation, rather than to replace clinical diagnosis or established care pathways. Regarding the scope of the GBA1 component, specifically, the proposed score applies to confirmed heterozygous carriers of pathogenic GBA1 variants only. Biallelic GBA1 mutations causing overt Gaucher disease are outside the scope of this framework because they represent a distinct clinical syndrome with different natural history, management requirements, and penetrance patterns. Patients with biallelic GBA1 mutations who receive enzyme replacement therapy for Gaucher disease and subsequently develop PD represent a further distinct subgroup not captured by the current PVI structure.

The variables listed in Table 2 are candidate variables for future prospective model development. The illustrative weights assigned to each variable reflect the current relative maturity of their evidence base rather than any empirical optimisation, and they serve only to organise the candidate variables into a provisional research framework. The final composition, weighting, and threshold structure of any validated vulnerability model must be determined by prospective data analysis, not by the qualitative judgements made in this review.

A specific rationale for each component selection is provided here for each component. GBA1 genotype was selected as the primary genetic vulnerability marker because it is the most prevalent genetic risk factor for sporadic PD, with established biological plausibility through lysosomal GCase deficiency, and because genotyping is already part of research protocols and increasingly available clinically. Point values of 3 (high-risk pathogenic variant) and 2 (lower-risk pathogenic variant) reflect the documented difference in relative risk and lysosomal enzyme deficit between variant classes (L444P-like variants: greater GCase deficit, higher PD risk; N370S-class: partial GCase deficit, lower but significant PD risk); these are evidence-informed relativities, not statistically derived coefficients. LRRK2 G2019S was included at 2 points (same tier as lower-risk GBA1) because it confers a comparable magnitude of PD risk through a distinct but intersecting lysosomal and vesicular trafficking mechanism, and because GBA1-LRRK2 co-occurrence is associated with additive risk. GCase enzymatic activity (CSF and DBS) was selected as the functional vulnerability marker because it provides a direct enzymatic readout of lysosomal capacity (the mechanism through which GBA1 variants produce neuronal vulnerability) and because reduction below published thresholds is associated with Lewy pathology burden and disease severity independent of GBA1 genotype. The specific thresholds (CSF: 39 nmol/h/mL from Parnetti et al. 2014; DBS: 2.8 nmol/h/mg from Lerche et al. 2021) were selected from the largest and most methodologically rigorous studies available at the time of this review; they are platform-specific research anchors, not universal cut-offs. Alpha-synuclein SAA was selected as the pathology marker because it is the most sensitive and specific available measure of active alpha-synuclein seeding in living patients, validated in the PPMI cohort and replicated across independent centres.

A single point was assigned because SAA positivity is a dichotomous state marker (present/absent) rather than a graded vulnerability score. DAT-SPECT was selected as the neurodegeneration marker because it is the established clinical neuroimaging standard for dopaminergic neuronal loss in PD, validated across multiple cohorts with quantitative striatal binding ratio thresholds, and because it provides an independent structural measure complementary to the functional and molecular markers above. MoCA and UPSIT were selected as clinical state variables because cognitive screening and olfactory function testing are among the most reproducible clinical indicators of PD subtype severity and are already part of standard research assessment batteries. Their inclusion in the PVI reflects the clinical reality that vulnerability biology expresses itself partly through these measurable functional outcomes.

Their limitation as disease-state rather than pure vulnerability markers is acknowledged explicitly in Section 5.2 of this revision. The overall point weighting structure (maximum 3 for highest-risk genetic variants; 1 for each functional/pathological/clinical marker) was constructed to weight cell-intrinsic genetic vulnerability highest, consistent with the hypothesis that biological vulnerability amplifies the impact of alpha-synuclein pathology rather than determining it independently. This is a theoretical weighting rationale, not an empirical one, and the relative weights must be tested and recalibrated in prospective cohort data before the PVI can function as a predictive tool.

In the specific context of GCase activity and alpha-synuclein SAA, the thresholds cited in this review (DBS GCase: 2.8 nmol/h/mg protein; CSF GCase: 39 nmol/h/mL, from Lerche et al. 2021 and Parnetti et al. 2014, respectively) are derived from single studies using specific assay platforms and must not be treated as universal cut-offs applicable across laboratories. Additionally, peripheral DBS GCase activity does not directly measure brain lysosomal GCase function; it is a surrogate marker whose relationship to neuronal lysosomal capacity has not been independently validated in post-mortem brain tissue.

For the SAA variable, only validated CSF-based assays should be used; blood-based and EV-derived assay platforms are methodologically heterogeneous, not yet equivalently validated, and must not be substituted for CSF SAA in the context of this framework without independent platform-specific comparative validation. Points for GCase and SAA in the candidate variable framework should therefore be applied only within laboratory settings that have established local reference ranges using the same assay conditions as the source studies.

Most components of the proposed score are already used in clinical or research neurology settings: genetic testing, dried blood spot or CSF GCase activity, alpha-synuclein SAA, MoCA, olfactory testing, and dopaminergic imaging. The novelty lies in combining these measures according to vulnerability biology. This also creates the main limitation: the combined score has not yet been validated, calibrated or externally replicated.

For this reason, the PVI is presented here as a candidate variable framework for prospective model development, not a validated scoring tool (as stated in Section 2.7). The score should not be used as the basis for individual patient management, genetic disclosure, treatment selection or advance care planning outside a research protocol until prospective validation has been completed.

A critical distinction must be maintained: the PVI is a conceptual vulnerability framework, not a predictive scoring tool. A validated predictive scoring tool derives coefficients from a fitted dataset with internal and external validation. The PVI does not meet this standard: its point values are illustrative weights reflecting evidence maturity, not validated regression coefficients, and its risk bands are proposed research categories for prospective testing, not clinically validated thresholds.

The selected biomarkers, cut-off values, point assignments, and risk categories are drawn from separate studies rather than from a single validated dataset, a fundamental limitation that must be stated prominently. A validated predictive scoring tool requires: biomarker selection and threshold optimisation on a training dataset; assessment of internal validity (e.g., cross-validation, bootstrap resampling); and replication on an independent external dataset. The PVI has undergone none of these steps.

Its individual components are each supported by independent evidence, but the combination of those components into a composite score has not been tested against clinical outcomes in any dataset. This does not make the framework arbitrary or invalid as a hypothesis-generating tool; it means its claims must be strictly limited to what the evidence supports: a structured proposal for prospective investigation, not a tool for individual risk prediction or clinical decision-making.

The relationship between any of these candidate variables and clinical outcomes is likely to be substantially modified by factors that the current framework does not model: age at onset and disease duration (both of which independently predict cognitive decline); disease stage at the time of assessment (later-stage patients will systematically score higher on disease-state markers regardless of intrinsic vulnerability); ancestry (GBA1 allele frequencies, GCase activity reference ranges, and olfactory normative data all vary between populations); prior or concurrent treatment (dopamine replacement therapy, cholinesterase inhibitors, and other medications modify cognitive and motor test performance); cognitive reserve (education, occupational complexity, and bilingualism modulate the relationship between neuropathological burden and cognitive test scores); and outcome definition (dementia, motor progression rate, falls, institutionalisation, and mortality are each distinct endpoints with different biological predictors).

Any future prospective model incorporating these candidate variables must pre-specify which outcome is being predicted, include the relevant modifiers as covariates, and be validated independently across populations with different ancestry and healthcare contexts before clinical implementation can be considered.

### 5.1. Relationship to Existing Biological Staging Frameworks

The PVI is not proposed as a competing disease definition. SynNeurGe and NSD-ISS have already moved PD research towards biological classification by incorporating alpha-synuclein status, neurodegeneration, genetic information and clinical features [7,8]. The PVI has a different purpose. It asks whether the biological vulnerability of neuronal systems, especially lysosomal, energetic, synaptic and inflammatory reserves, can help predict progression and enrich future trials within biologically defined PD cohorts.

In practical terms, the PVI should be considered complementary to these frameworks. A patient could be classified biologically by SynNeurGe or staged by NSD-ISS, then further stratified within a research cohort by a vulnerability score. Whether this additional layer improves prediction beyond existing biological staging remains an empirical question and is one of the central validation tasks proposed by this review.

### 5.2. PVI Scoring

Cut-off sources and measurement anchors include GCase source studies, SAA studies, MoCA validation work and UPSIT normative testing (Table 3) [48,49,51,52,57,58].

To assist in the interpretation of Table 2, the category of each PVI component is explicitly listed here. Vulnerability markers (cell-intrinsic biological properties assessable before pathology is established): GBA1 genotype (severe allele, mild allele, LRRK2 G2019S, co-occurrence), CSF GCase activity, dried blood spot GCase activity. Pathology marker (reflects active alpha-synuclein seeding): CSF alpha-synuclein SAA. Neurodegeneration marker (reflects established neuronal or synaptic loss): DAT-SPECT striatal binding. Clinical state variables (current functional status at a single timepoint): MoCA score at diagnosis, UPSIT olfactory function. Longitudinal outcome variables (require serial assessment; cannot be scored at baseline): 12-month MoCA decline (≥ 2 points), informant-corroborated cognitive concern emerging during follow-up. This mapping confirms that the PVI is not a homogeneous biological construct; it combines measures from five distinct domains differing in biological meaning, temporal scope, and evidence type.

Any validation study must test the independent predictive contribution of each category before the composite score can be meaningfully interpreted. An important structural limitation requires explicit acknowledgement: MoCA score, olfactory function (UPSIT), and dopaminergic imaging (DAT-SPECT) reflect current disease state and established neuronal loss rather than intrinsic neuronal vulnerability, potentially introducing circularity into the predictive model: a patient with more advanced disease may score highly partly because of existing degeneration. Prospective validation must explicitly test whether vulnerability markers (GBA1, GCase, SAA) or disease-state markers (MoCA, UPSIT, DAT-SPECT) drive predictive performance; if the latter predominate, the PVI would offer limited advantage over existing staging tools.

To explain the circularity concern more fully, the PVI is proposed as a tool for predicting future disease trajectory, specifically to identify patients at highest risk of cognitive decline, non-motor progression, and dementia. Circularity arises when the predictor variables already reflect the very outcome being predicted. In this case, MoCA score, UPSIT, and DAT-SPECT striatal binding all reflect the degree of existing neuronal degeneration and functional impairment at the time of assessment. Since the primary outcome of interest, future cognitive decline, correlates strongly with baseline cognitive status, olfactory function, and dopaminergic degeneration, a model that includes these three variables will inevitably appear to predict future cognitive decline even without any genuine vulnerability biology signal. In statistical terms, including disease-state markers as predictors when they correlate strongly with the outcome inflates apparent predictive performance and makes it impossible to determine whether any additional signal comes from the vulnerability biology or simply from the severity of existing disease at baseline.

There are two distinct forms of circularity that the PVI may introduce. The first is cross-sectional circularity: Higher PVI scores at any timepoint will correlate with more advanced disease at that timepoint, not because the PVI captures vulnerability biology but because three of its seven components directly measure disease severity. This means the PVI cannot be used as a cross-sectional staging tool without it becoming equivalent to an existing staging measure, which would undermine its claim to add new information. The second is predictive circularity: If a patient already has a low MoCA, impaired olfaction, and reduced DAT binding at baseline, predicting future cognitive decline from these variables is partially tautological; the outcome is already partially manifested at the time of prediction.

Whether circularity is actually introduced depends on how the PVI is used. If the PVI is used to predict future clinical outcomes from baseline assessment in newly diagnosed patients, and if MoCA, UPSIT, and DAT-SPECT scores at diagnosis reflect starting-point disease severity rather than trajectory, then a degree of circularity is introduced but may not be fatal to the framework; it would reduce the incremental value of the vulnerability biology markers rather than invalidating them.

If, however, the PVI is used to enrich clinical trial populations for patients with high-vulnerability biology, the disease-state markers should not drive the enrichment criterion, because that would simply select patients with more advanced disease at baseline, which existing staging tools already do. In this context, combining vulnerability markers with disease-state markers in a single composite score is scientifically problematic because it conflates two different types of information that serve different purposes in trial design. The circularity concern can be addressed by the following validation design principles, which should be pre-specified before any prospective validation study of the PVI. First, the vulnerability markers (GBA1 genotype, GCase activity, SAA status) and the disease-state markers (MoCA, UPSIT, DAT-SPECT) should be analysed in separate models before being combined to establish their independent predictive contributions.

Second, the incremental predictive value of adding the vulnerability markers to a baseline model containing only the disease-state markers should be formally tested (e.g., using likelihood ratio tests, C-statistic improvement, net reclassification improvement); if the incremental value is negligible, the PVI provides no advantage over existing staging. Third, for trial enrichment applications, specifically, the vulnerability markers alone (without disease-state markers) should be evaluated as the enrichment criterion to avoid selecting patients on the basis of existing severity rather than biological mechanism. Fourth, where technically and ethically feasible, vulnerability markers should ideally be measured before significant neurodegeneration is established (e.g., in the prodromal or newly diagnosed stage), with disease-state markers used as longitudinal outcome variables rather than as baseline predictors.

These design principles would allow the genuine predictive contribution of vulnerability biology to be separated from the confounding influence of disease severity at baseline, and would provide a definitive test of whether the PVI adds value beyond existing staging.

The following variables represent changes that occur over time and require serial assessment. They are outcome measures to be predicted by the baseline PVI score (Modules 1–3), not components of the score itself. These measures should be used in prospective validation studies to test whether the baseline vulnerability and pathology markers predict future clinical progression. This separation addresses the temporal inconsistency and circularity concerns raised in the peer review process (Table 4).

These outcome measures are not scored as part of the PVI at baseline. They represent the clinical endpoints that the PVI framework aims to predict. Prospective validation studies should test whether the baseline PVI score (Modules 1–3) predicts these outcomes after adjustment for relevant covariates including age, disease duration, baseline disease severity, and ancestry.

Interpreting the Score in Research Settings: A Proposal for Empirical Derivation.

Important: The global risk bands presented in this framework (0–2, 3–5, ≥6) are proposed research categories for prospective testing, not validated prognostic thresholds. They are included solely to provide a structured basis for hypothesis-driven validation studies and mechanism-targeted trial enrichment protocols. The division into three bands reflects a qualitative heuristic based on the sum of candidate point weights, which themselves are illustrative and not statistically derived.

No clinical action should be taken on the basis of these unvalidated bands outside a formally approved validation study protocol.

For research purposes, we propose that any prospective validation study should:

Derive empirical risk bands empirically from the distribution of the composite score in the training dataset (e.g., using tertiles, quartiles, or optimal cut-point analysis), rather than applying the 0–2, 3–5, ≥6 thresholds a priori.

Evaluate the score as a continuous predictor in regression models before dichotomising or categorising, to avoid loss of information and arbitrary threshold effects.

Optimise point weights using multivariable regression, survival analysis, or machine learning approaches on a training dataset, with internal validation (cross-validation or bootstrap resampling) and external replication on an independent cohort.

Determine the incremental predictive value of the composite score over existing staging systems (SynNeurGe, NSD-ISS) and established covariates (age, disease duration, baseline motor severity, APOE genotype, amyloid/tau burden) before any categorisation is applied.

Until such empirical derivation and validation have been performed, the 0–2, 3–5, and ≥6 bands remain heuristic research proposals awaiting calibration, not evidence-based risk categories. Investigators using these bands in research protocols must treat them as starting-point hypotheses requiring local recalibration against the specific study population, assay platforms, and pre-specified clinical endpoints.

Table 4: Proposed research monitoring and trial-enrichment implications for each PVI risk band. All suggestions represent hypothetical research protocol examples and are NOT evidence-based clinical guidelines. Monitoring intervals and care planning suggestions are NOT supported by direct comparative evidence and must NOT be implemented in clinical practice. Abbreviations: ACP = advance care planning; GCase = glucocerebrosidase enzyme; LBD = Lewy body dementia; MoCA = Montreal Cognitive Assessment; PVI = Parkinson’s Vulnerability Index; RBD = REM sleep behaviour disorder; SAA = seed amplification assay.

A score of 6 or above, if validated prospectively and after adjustment for relevant modifiers, might identify a group with higher proposed biological vulnerability appropriate for enriched follow-up within research studies or screening for mechanism-targeted clinical trials. This must not be interpreted as a clinical prediction for any individual patient, and must not trigger treatment escalation, family cascade testing, or advance care planning outside standard clinical criteria and approved research protocols (Table 5). The three risk bands (0–2, 3–5, ≥6) in this framework are provisional research categories whose clinical utility has not been established.

They are included to provide a structured basis for hypothesis-driven validation studies and mechanism-targeted trial enrichment protocols, not as validated prognostic tools. Any investigator wishing to use these bands in a research study must treat them as starting-point hypotheses requiring local recalibration against the specific study population, assay platforms, and pre-specified clinical endpoints.

These patterns are heuristic and require validation against longitudinal biomarker cohorts (Table 6).

**Figure 4:**
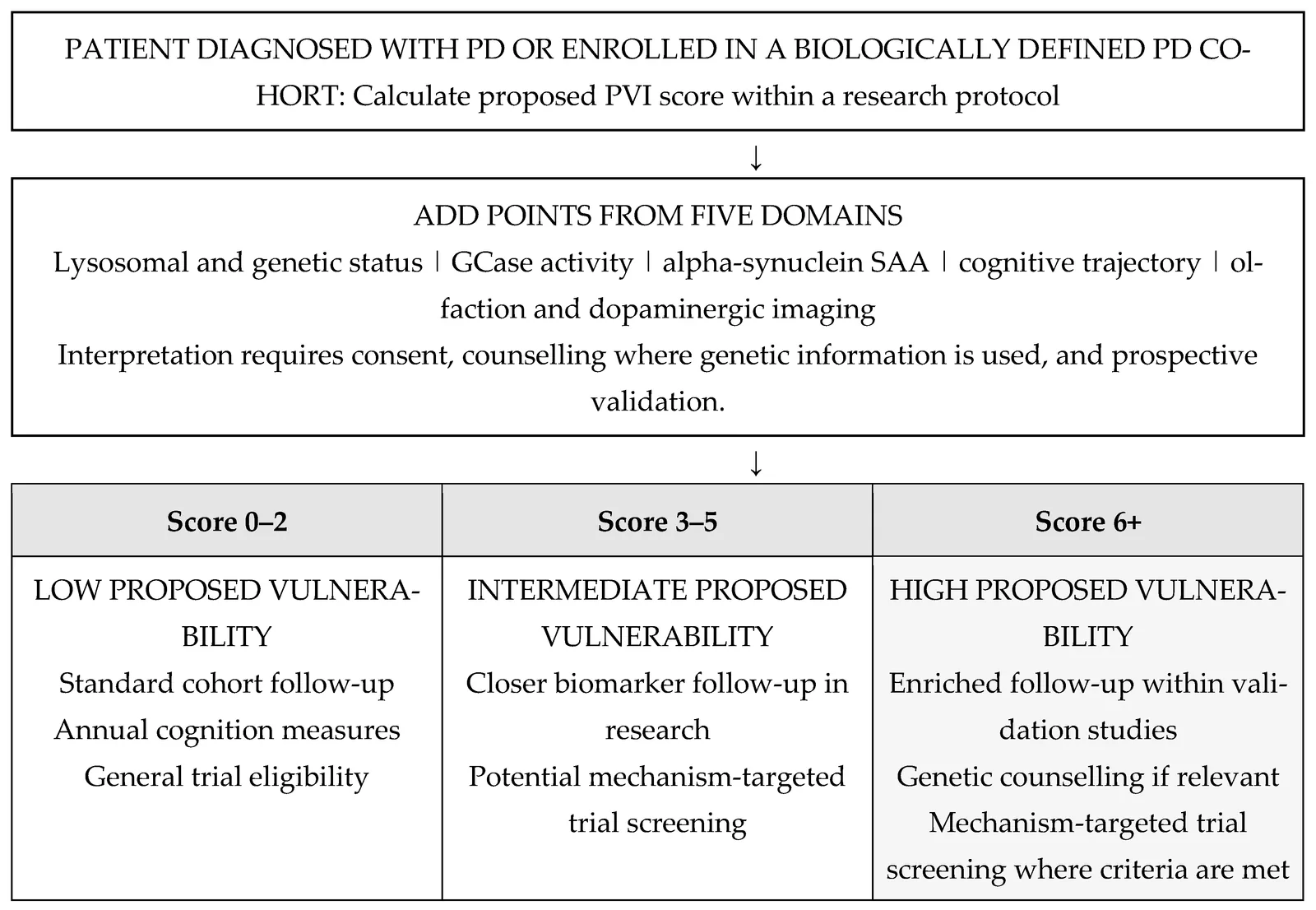
Proposed PVI research decision pathway at diagnosis using baseline modules only. The figure illustrates the proposed algorithmic decision pathway for applying the Parkinson’s Vulnerability Index (PVI) in a biologically defined research cohort. Patients first have a baseline PVI score calculated by summing points from Module 1 (vulnerability biomarkers: GBA1 genotype, LRRK2 G2019S genotype, CSF GCase activity, DBS GCase activity), Module 2 (pathology and staging biomarkers: CSF alpha-synuclein SAA, DAT-SPECT), and Module 3 (baseline clinical measures: MoCA score, UPSIT olfactory function). Module 4 (prospective outcome measures: 12-month MoCA decline, informant-corroborated cognitive concern, motor progression, conversion to dementia) is NOT included in the baseline score; these variables are outcomes to be predicted in longitudinal validation studies. The total baseline score places the patient into one of three proposed research categories (0–2, 3–5, ≥6) that have NOT been empirically derived or validated. These bands are heuristic proposals for prospective testing; their clinical utility remains unproven. This pathway is intended exclusively for formally approved validation studies and trial-enrichment protocols; it must not be used for routine clinical decision making, individual prognosis, or treatment selection outside a research protocol. GCase = glucocerebrosidase; LBD = Lewy body dementia; MoCA = Montreal Cognitive Assessment; SAA = seed amplification assay.

The PVI score is calculated by adding points from vulnerability-related measurement domains. The total score places the patient into a proposed research risk band. This pathway is intended for validation studies and trial-enrichment protocols, not for routine clinical decision-making (Figure 4).

IMPORTANT: The three modes in this table are explicitly hypothetical heuristic illustrations pending prospective validation. The estimated patient proportions (~55–60%, ~20–25%, ~15–20%) are speculative placeholders with no statistical derivation and must NOT be cited as established prevalence estimates. The association between specific PVI risk bands and these progression modes has not been empirically tested and remains a hypothesis for future validation studies.

## 6. Discussion

### 6.1. Why Neuroprotective Trials Have Failed, and How to Fix Them

Several neuroprotective trials in PD have failed to show convincing disease-modifying efficacy in broad, clinically defined populations. The field is now more nuanced than a simple failure narrative: prasinezumab did not meet its original primary endpoint, but subsequent exploratory and extension analyses suggested possible motor effects in faster-progressing subgroups [59,60,61]. Lixisenatide showed less motor progression over 12 months in a phase 2 trial, although larger and longer studies are needed [62]. Ambroxol studies provide evidence of safety and target engagement in GCase biology, while disease-modifying effects in GBA1-related PD remain under investigation [63,64]. Earlier creatine, coenzyme Q10 and cinpanemab trials further illustrate the difficulty of demonstrating disease modification in heterogeneous PD populations [65,66,67].

One explanation is that many trials enrolled by diagnosis rather than by the biological mechanism active in each participant. A drug targeting lysosomal dysfunction is unlikely to benefit all patients with PD if only a subset has measurable lysosomal impairment. The same problem applies to mitochondrial, inflammatory or anti-aggregation strategies when the enrolled population is biologically heterogeneous.

The prasinezumab programme illustrates the importance and the risk of subgroup interpretation. The overall early-stage PD trial did not meet its primary outcome, but later analyses suggested that patients with features of faster progression may show a larger motor signal [59,60,61]. This finding is hypothesis-generating rather than definitive. It nonetheless supports the broader principle that neuroprotective trials may need to pre-specify biologically or clinically enriched subgroups rather than rely on broad diagnostic enrolment.

The implication for future trial design is practical but still needs validation. GCase-targeted trials could pre-specify enrichment or stratification by GBA1 status, GCase activity and alpha-synuclein SAA status [63,64]. Incretin or metabolic trials could similarly examine whether baseline inflammatory, metabolic or progression-rate markers identify responder groups [62]. A vulnerability-based framework such as the PVI could help organise these questions, but only after it has been tested against existing staging systems and longitudinal outcomes.

### 6.2. What Still Needs to Be Done

Despite the strength of the mechanistic rationale, several gaps prevent the PVI from being considered a clinical standard. These gaps are not minor technical issues; they define the research programme that would be needed before vulnerability-based stratification could be used safely and meaningfully.

The most fundamental gap is that we know much more about why certain nerve cells die than why others survive. The VTA dopamine neurons, the calbindin-positive SNpc cells, and cerebellar Purkinje cells all resist alpha-syn pathology that destroys their neighbours, and their protective biology is almost entirely unstudied. A large-scale molecular comparison of surviving and dying neurons from PD post-mortem brain tissue, controlling for disease stage and genetic background, would be transformative.

Second, the PVI requires prospective composite validation (methodological limitations are addressed in Section 6.4); such work could be embedded into large cohort studies such as PPMI, with pre-specified endpoints including conversion to PD dementia, clinically meaningful MoCA decline, motor progression, SAA status, DAT imaging change, institutionalisation and mortality [68]. A realistic validation period would be five to seven years, depending on baseline disease stage and event rates.

A validation study should test whether the PVI adds predictive value beyond age, disease duration, baseline motor severity, MoCA, SAA status, DAT imaging and genetic status alone. Statistical evaluation should include discrimination, calibration, internal validation, external replication and decision curve analysis. The score should also be tested for fairness across sex, ancestry, age at onset and access to biomarker testing.

Essential covariates for PVI validation: When cognitive progression is the primary endpoint as it is for many PVI applications, additional covariates must be included in any validation model. Even though amyloid-beta, tau, TDP-43 pathology, APOE genotype, and vascular burden are not included as core vulnerability domains in the proposed PVI, they are well-established modifiers of cognitive trajectory in PD and must be incorporated as essential covariates in any prospective validation of the framework. Specifically:

(a) Amyloid-beta pathology. CSF Aβ42 or Aβ42/Aβ40 ratio, or amyloid PET imaging, should be included as a covariate because amyloid co-pathology is associated with faster cognitive decline in PD and may confound the relationship between vulnerability markers and cognitive outcomes.
(b) Tau pathology. CSF p-tau181, p-tau217, or tau PET imaging should be included because tau burden correlates with cognitive impairment and dementia risk in PD, independent of alpha-synuclein pathology.
(c) TDP-43 pathology. While less readily measurable in living patients, TDP-43 co-pathology is common in older PD patients and contributes to cognitive decline; when available, TDP-43 biomarkers (e.g., CSF TDP-43) should be included as covariates.
(d) APOE genotype. APOE ε4 status should be included as a covariate because it is the strongest genetic predictor of cognitive decline in the general population and modifies the relationship between amyloid pathology and cognitive outcomes in PD.
(e) Vascular burden. White matter hyperintensity burden on MRI, history of cerebrovascular disease, and vascular risk factors (hypertension, diabetes, hyperlipidaemia) should be included because vascular pathology contributes to cognitive impairment independently of neurodegenerative pathology.
(f) Co-occurring proteinopathies more broadly. As noted in Section 2.6, co-occurring proteinopathies (amyloid-beta, tau, TDP-43) were excluded from the primary vulnerability domains because they represent extrinsic pathological co-occurrences that modify clinical expression rather than cell-intrinsic properties determining susceptibility to alpha-synuclein pathology specifically. However, this exclusion does not diminish their importance; they must be included as covariates and effect modifiers in any validation study where cognitive progression is the outcome of interest.

The rationale for including these covariates is that they may confound the relationship between vulnerability markers and cognitive outcomes. For example, a patient with high PVI score may also have high amyloid burden; without controlling for amyloid, the PVI might appear to predict cognitive decline when in fact the amyloid burden is the primary driver. Similarly, APOE ε4 status modifies the relationship between amyloid pathology and cognitive outcomes and should therefore be included as a covariate rather than as a component of the vulnerability score.

Analytical approach for covariate adjustment: We recommend that validation studies test three nested models in addition to the models described in Section 5.2: Model 4—baseline modules (1+2+3) + amyloid/tau/APOE covariates; Model 5—baseline modules (1+2+3) + vascular burden covariates; Model 6—all covariates combined. Comparing these models will determine whether the PVI adds predictive value beyond established cognitive modifiers and whether the vulnerability markers and the co-pathology covariates make independent contributions to prediction. If the PVI’s predictive performance is substantially attenuated by the addition of these covariates, this would suggest that the vulnerability markers are not independent predictors of cognitive trajectory and that cognitive progression in PD is primarily determined by co-pathology burden rather than by cell-intrinsic vulnerability to alpha-synuclein. Only after such analyses can the independent contribution of vulnerability biology to cognitive outcomes be properly evaluated.

Third, laboratory models of PD vulnerability currently focus almost entirely on SNpc dopamine neurons. No published study has compared how dopaminergic, noradrenergic, cholinergic, and cortical neurons from the same patient respond to identical alpha-syn exposure under controlled conditions. This comparison would directly answer questions about cell-intrinsic vulnerability that post-mortem studies cannot. Fourth, the role of astrocytes (supportive brain cells) in selective vulnerability has been largely overlooked compared with microglial research. Fifth, most vulnerability studies exclude patients with mixed brain pathologies (combining PD with Alzheimer’s-type or TDP-43 pathology), yet mixed pathology is the most common finding in older PD patients. This exclusion introduces a systematic bias that should be addressed in dedicated studies.

### 6.3. Vulnerability Biology, Propagation, and Clinical Trajectory

This review argues that the clinical heterogeneity of PD can be understood more clearly when propagation biology is considered alongside selective neuronal vulnerability. Alpha-synuclein exposure matters, but the response of each neuronal population depends on its calcium burden, energetic reserve, lysosomal capacity, axonal architecture and inflammatory microenvironment. This provides a biologically coherent explanation for why the same pathological protein can produce different clinical trajectories.

This framing keeps propagation biology firmly in view. Alpha-synuclein spread helps define the geography of exposure. Vulnerability biology helps explain what happens after exposure. This distinction is important because therapies that only block propagation may be insufficient if neurons are already energetically depleted, lysosomally impaired or exposed to a damaging inflammatory environment.

The GBA1 story provides the clearest example of this principle. The mechanism, biomarker evidence, and clinical heterogeneity of GBA1-PD are fully detailed in Section 4, substantially between cohorts, variant types, and ancestral backgrounds. GBA1-PD should not be described as a separate disease entity: it represents a subgroup with modified probabilistic risk within the PD spectrum, and individual outcomes remain incompletely predictable even within this subgroup.

The comparison with related synucleinopathies reinforces the importance of cellular context. In multiple-system atrophy, alpha-synuclein accumulates predominantly in oligodendrocytes rather than neurons, producing a different disease architecture [69]. In dementia with Lewy bodies, earlier cortical involvement and different clinicopathological correlations suggest that cortical vulnerability and network context shape the phenotype [70]. The same pathological protein can therefore produce different syndromes depending on the cell type, region and biological environment in which it accumulates.

The role of co-occurring proteinopathies in modifying PD progression: An important consideration in any discussion of cognitive trajectory in PD is that most older patients with PD have co-occurring proteinopathies in addition to alpha-synuclein pathology. Post-mortem studies consistently demonstrate that a substantial proportion of PD patients have concomitant amyloid-beta plaques (approximately 30-50%), tau neurofibrillary tangles (approximately 40–60%), or TDP-43 pathology (approximately 20-40%) at autopsy. These co-pathologies are not simply epiphenomena; they are independently associated with cognitive decline and dementia risk in PD. The presence of amyloid-beta pathology, in particular, is associated with faster cognitive decline and higher dementia risk, while tau burden correlates with the severity and rate of cognitive impairment. TDP-43 pathology has been linked to more rapid cognitive decline and higher dementia risk in PD, independent of Alzheimer’s-type pathology. Vascular burden, including white matter hyperintensities and lacunar infarcts, also contributes to cognitive impairment and may interact synergistically with neurodegenerative pathology. This has profound implications for the interpretation of vulnerability markers. A patient with high vulnerability to alpha-synuclein pathology (e.g., GBA1 carrier with low GCase activity) may also have high amyloid or tau burden, and the cognitive decline observed in that patient may be driven by co-pathology rather than by alpha-synuclein vulnerability alone. Without controlling for these co-pathologies in validation studies, the independent contribution of vulnerability markers to cognitive outcomes cannot be properly evaluated. This is why we emphasise—in Section 2.6 and Section 5.2—that amyloid, tau, TDP-43, APOE, and vascular burden must be included as essential covariates in any prospective validation of the PVI, even though they are not included as core vulnerability domains. The PVI should therefore be seen as part of a broader multi-marker approach to predicting PD progression, rather than as a standalone prognostic tool.

Seen through this framework, the failure of many neuroprotective trials should be interpreted cautiously. Some targets may have been biologically relevant but tested in insufficiently stratified populations. The next step is not simply to abandon these mechanisms, but to test them in cohorts where the target pathway is demonstrably active and where biomarkers can confirm target engagement.

#### Digital Biomarkers and Artificial Intelligence: The Next Frontier in Vulnerability Stratification

The future evolution of vulnerability-based stratification in Parkinson’s disease will likely depend not only on molecular biomarkers but also on the integration of digital biomarkers capable of continuously capturing subtle functional changes during the earliest stages of disease progression. Advances in wearable technologies, smartphone-based assessments, passive home monitoring, and remote sensing now enable objective measurement of gait dynamics, balance, tremor characteristics, speech alterations, typing behaviour, sleep architecture, and daily physical activity under real-world conditions. Unlike conventional clinical assessments, which provide only intermittent snapshots of disease status, digital biomarkers generate high-frequency longitudinal data that may reveal dynamic changes in neuronal function before measurable structural neurodegeneration becomes apparent.

Importantly, these technologies should not be viewed as replacements for biological biomarkers such as GBA1 genotype, glucocerebrosidase activity, α-synuclein seed amplification assays, or neuroimaging, but rather as complementary measures that capture the functional consequences of underlying biological vulnerability. Continuous monitoring of motor and non-motor performance may improve the identification of patients with accelerated disease trajectories and facilitate more precise phenotypic characterisation within biologically defined subgroups.

The multidimensional nature of the proposed Parkinson’s Vulnerability Index also highlights the growing importance of artificial intelligence (AI) and machine learning approaches. Integrating genomic, biochemical, neuroimaging, digital, and clinical data into a unified predictive framework exceeds the capabilities of conventional statistical models. Machine learning algorithms offer the opportunity to identify complex nonlinear interactions among vulnerability domains, optimise biomarker weighting, and generate individualised risk profiles based on multimodal datasets. Future prospective cohort studies incorporating biological, imaging, and digital biomarkers may therefore provide the empirical foundation necessary to transform the current conceptual framework into a validated precision medicine platform.

Although digital biomarkers remain under active validation and important challenges regarding standardisation, interoperability, data privacy, and clinical implementation persist, they represent one of the most promising avenues for future research. Their integration with molecular biomarkers may ultimately enable continuous, non-invasive monitoring of neuronal vulnerability, improve patient stratification for disease-modifying clinical trials, and support the development of truly personalised therapeutic strategies in Parkinson’s disease.

### 6.4. Limitations

This review should be interpreted in light of several important limitations. First, despite employing a structured search strategy with predefined eligibility criteria, independent screening by two reviewers, and transparent evidence selection, the present work remains a structured critical narrative review rather than a systematic review or meta-analysis. Consequently, the synthesis is inherently qualitative and does not provide pooled effect estimates, quantitative measures of heterogeneity, or formal comparative assessments of evidence strength across studies.

Second, the literature included in this review is characterised by substantial methodological heterogeneity. The available evidence encompasses human post-mortem investigations, genetic association studies, biomarker research, neuroimaging studies, induced pluripotent stem cell models, animal experiments, and longitudinal clinical cohorts. These study designs differ considerably with respect to patient selection, disease stage, experimental methodology, outcome definitions, and biomarker measurement techniques, limiting direct comparisons and precluding quantitative synthesis.

Third, although methodological quality was critically considered during evidence appraisal, no formal risk-of-bias instrument (such as RoB 2, ROBINS-I, or the Newcastle-Ottawa Scale) was applied because of the narrative design and the diversity of included study types. Consequently, the relative contribution of individual studies to the overall conclusions inevitably reflects expert interpretation, introducing a degree of subjectivity that cannot be entirely eliminated.

Fourth, the five vulnerability domains proposed in this review were identified through an iterative evidence-synthesis process rather than through a formal consensus methodology or data-driven statistical framework. To enhance transparency, we have documented in Section 2.6 the assessment of each candidate mechanism against the three pre-specified criteria (reproducibility across biological platforms, mechanistic relevance to selective neuronal vulnerability, and translational potential), along with the justification for the final classification decision (included, nested within an existing domain, or excluded). This transparent audit trail allows readers to evaluate the evidentiary basis for our domain selection. Nevertheless, the final selection remains partially dependent on expert judgement. Alternative conceptual classifications or weighting of biological mechanisms may therefore be equally plausible, and future Delphi-based consensus studies or quantitative evidence-mapping approaches could further refine this framework.

An additional limitation concerns the translational maturity of currently available biomarkers. While several candidate markers, particularly GBA1 genotype, glucocerebrosidase activity, and cerebrospinal fluid α-synuclein seed amplification assays, show considerable promise, many proposed biomarkers remain incompletely standardised. Differences in assay platforms, laboratory protocols, pre-analytical processing, reference ranges, and threshold definitions reduce comparability across studies and currently limit immediate implementation in routine clinical practice.

Furthermore, the proposed Parkinson’s Vulnerability Index (PVI) should be regarded exclusively as a hypothesis-generating research framework. The proposed variables, domain weights, and threshold values were derived from biological plausibility and the current literature rather than from prospective multivariable modelling or statistical optimisation. Accordingly, the PVI has not undergone external validation, calibration, discrimination testing, or assessment of incremental prognostic value relative to established biological staging systems such as SynNeurGe or the NSD-ISS. Its clinical utility therefore remains entirely unproven.

Additionally, the absence of established covariates in the current PVI framework represents a significant limitation that must be acknowledged. While the PVI focuses on cell-intrinsic vulnerability mechanisms, it does not currently incorporate well-established modifiers of cognitive trajectory including amyloid-beta pathology, tau pathology, TDP-43 co-pathology, APOE genotype, and vascular burden. These factors are known to substantially modify the clinical expression of PD, particularly cognitive decline and dementia risk, and their omission from the PVI score means that the framework cannot currently account for their confounding effects. This is a deliberate design choice these factors were excluded from the core vulnerability domains because they represent extrinsic co-pathologies rather than cell-intrinsic properties but it is nonetheless a limitation that must be transparently communicated. Any future prospective validation of the PVI must include these factors as essential covariates, as detailed in Section 5.2. Without such adjustment, the independent contribution of vulnerability markers to cognitive outcomes cannot be properly evaluated. The PVI should therefore be understood as a framework that complements, rather than replaces, existing knowledge about other determinants of disease progression. Future iterations of the PVI should consider whether some of these covariates should be incorporated into an expanded framework, particularly as biomarkers for amyloid, tau, TDP-43, and vascular burden become more widely available and standardised.

Finally, Parkinson’s disease is a biologically complex and multidimensional disorder, and additional mechanisms including epigenetic regulation, ferroptosis, neurovascular dysfunction, DNA damage responses, systemic immune interactions, and concomitant proteinopathies may contribute significantly to neuronal vulnerability. These mechanisms were intentionally not incorporated as independent domains because current evidence does not yet support robust patient-level biomarker stratification. As knowledge of PD biology evolves, future iterations of the proposed framework will likely require modification to incorporate newly validated molecular pathways and biomarkers.

An additional limitation that warrants explicit emphasis concerns the provisional nature of the global PVI risk bands (0–2, 3–5, ≥6). Although we have stated throughout this review that the point weights and thresholds are illustrative and not statistically derived, we acknowledge that the continued presentation of these bands as ‘low,’ ‘intermediate,’ and ‘high’ may inadvertently convey a degree of quantitative validity that is not currently supported by evidence. These bands are heuristic research proposals awaiting empirical derivation and validation; they should not be interpreted as evidence-based risk categories. Any future validation study must derive risk bands empirically from the distribution of the composite score in the training dataset, optimise weights using appropriate statistical methods, and validate the score on an independent external cohort before any categorisation is applied. Until such work has been completed, the proposed bands should be understood exclusively as hypothesis-generating tools for research design, not as validated prognostic instruments.

Despite these limitations, the present review provides a biologically integrated synthesis of current evidence regarding selective neuronal vulnerability in Parkinson’s disease and proposes a transparent conceptual framework intended to stimulate prospective validation studies. Future multicentre longitudinal cohorts integrating multimodal biomarkers, standardised laboratory methodologies, and advanced computational modelling will be essential to determine whether vulnerability-based stratification can improve prognostic assessment and facilitate precision medicine approaches in Parkinson’s disease.

### 6.5. Ethical Considerations and Equity

The ethical dimension is equally important. A high vulnerability score could affect anxiety, family planning, insurance concerns, trial access and prognostic conversations. Any future implementation would require pre-test counselling, transparent communication of uncertainty, equitable access to testing and safeguards against using an unvalidated score to restrict or prioritise care. For now, the PVI is best understood as a framework for research, validation and trial design.

More specifically: incomplete penetrance means most variant carriers will not develop the predicted clinical outcome individually; incidental findings have implications for relatives who have not consented to testing; variants of uncertain significance require structured expert reclassification before disclosure; pre-test and post-test genetic counselling by appropriately trained personnel is a prerequisite; access to biomarker testing is unequal globally; olfactory testing requires culturally appropriate normative data; and GCase assay thresholds derived predominantly from European cohorts require recalibration for populations with different GBA1 allele frequencies.

Prospective validation must include diverse populations, ancestry-stratified reference ranges, and a commitment to equitable access. Access disparities represent a fundamental ethical concern that the manuscript must address directly and prominently. The five PVI components include interventions and tests with substantially different availability and cost profiles across healthcare systems. Genetic testing for GBA1, LRRK2, and PINK1/PARKIN is increasingly available in high-income settings but is not routinely accessible in low- and middle-income countries, where the majority of the world’s PD population resides.

A further equity consideration concerns the covariates proposed for PVI validation. The inclusion of amyloid, tau, TDP-43, and APOE biomarkers in validation studies raises additional access barriers. CSF collection for amyloid and tau biomarkers requires lumbar puncture, an invasive procedure with limited availability in many settings. Amyloid and tau PET imaging requires access to cyclotron facilities for radiotracer production, specialised PET infrastructure, and substantial financial resources that are unavailable in most healthcare systems globally. APOE genotyping is relatively accessible but raises the same genetic counselling and cascade testing concerns as GBA1 and LRRK2 testing. Vascular burden assessment by MRI requires access to neuroimaging infrastructure that is also unevenly distributed. The requirement to include these covariates in validation studies therefore creates a tension: scientifically necessary for valid inference, but practically inequitable in its access implications. This tension must be explicitly addressed in study design. Validation studies should consider whether less invasive or more accessible proxy measures (e.g., plasma p-tau217 as a proxy for amyloid and tau pathology, or vascular risk factor scores as a proxy for vascular burden) could serve as adequate covariates while maintaining feasibility across diverse settings. However, the use of proxy measures requires independent validation before they can be confidently substituted for gold-standard biomarkers. This is an area requiring careful methodological development.

Cerebrospinal fluid collection by lumbar puncture is an invasive procedure associated with patient burden, procedural risk, and technical demands that limit its use in many settings; its routine use for GCase measurement or SAA testing must not be assumed to be feasible across all clinical environments. Alpha-synuclein SAA testing requires specialist laboratory infrastructure and standardised quality control procedures that are concentrated in academic centres in high-income countries; a research framework proposing SAA as a scored component must acknowledge that this creates an inherent access inequality in who can participate in validation studies and in who can benefit from any future stratified care approach.

Dopamine transporter SPECT (DAT-SPECT) is a nuclear imaging investigation that requires a cyclotron facility for radiotracer production, licenced nuclear medicine infrastructure, radiation protection procedures, and specialist reporting. In many health systems, even within high-income countries, it is not universally available and is associated with substantial cost. Proposing DAT-SPECT as a routine scored component of a vulnerability index raises significant equity concerns: a scoring framework that assigns points for access to expensive investigations will systematically produce higher scores in patients with better healthcare access, independent of their true biological vulnerability. This access-driven confounding must be explicitly acknowledged as a limitation and as a design priority for future validation studies. Ancestry-related bias in reference data and assay calibration is a second major equity concern that affects multiple components of the PVI framework.

GCase activity reference ranges and risk-associated thresholds have been established predominantly in European and Ashkenazi Jewish cohorts; the applicability of these thresholds to patients of African, Asian, Latin American, or any other ancestry has not been validated and cannot be assumed. GBA1 variant frequencies, allele compositions, and associated penetrance estimates differ substantially between ancestral populations, meaning that variant classification frameworks and risk estimates derived from one population may not apply to another.

The University of Pennsylvania Smell Identification Test (UPSIT), used for olfactory scoring in the PVI, was primarily developed and normed in North American populations of predominantly European ancestry. The 40 odorants used in the UPSIT include items (e.g., root beer, bubble gum, pizza) that are culturally specific and unfamiliar to test-takers from populations not exposed to these scents, introducing systematic bias against individuals from non-Western cultural backgrounds. This olfactory assessment bias is not only a technical issue but also a matter of scientific validity: lower UPSIT scores in culturally non-matched populations will falsely suggest greater vulnerability and inflate PVI scores in certain groups.

The alpha-synuclein SAA literature is also derived primarily from European and North American cohorts; whether assay performance characteristics (sensitivity, specificity, threshold for positivity) are equivalent across ancestral backgrounds has not been established. The need for validated local normative data is therefore not optional but scientifically essential for several PVI components. Olfactory function must be assessed using locally validated normative datasets that account for age, sex, and cultural background in the population being tested; use of UPSIT North American norms in non-Western populations is methodologically unjustified and should not be permitted in PVI validation studies. GCase activity reference ranges must be established locally using the specific assay platform, substrate, and normalisation method applied in each research centre, and must be calibrated in an ancestry-matched reference population.

DAT-SPECT striatal binding ratio normative data vary between camera types, reconstruction algorithms, and imaging protocols; locally validated age- and sex-matched reference ranges are required for any quantitative threshold to be applied with validity. Cognitive normative data for the MoCA similarly require local validation, since educational attainment, language background, and cultural context all influence performance on standard cognitive screening instruments independently of dementia-related pathology. The implications for biological relatives of patients receiving genetic testing as part of the PVI framework deserve fuller discussion than the brief mention above. When a pathogenic GBA1 or LRRK2 variant is identified, this result has direct implications for first-degree relatives (parents, siblings, children) who share a 50% probability of carrying the same variant.

These relatives have not consented to receiving genetic information and may not wish to know their carrier status. The cascade testing implications must be discussed with the patient as part of pre-test counselling, and results must be communicated in a way that respects both the patient’s right to know and the relatives’ right not to know. A GBA1 result also has implications for reproductive decision-making in patients of childbearing age, since biallelic GBA1 inheritance causes Gaucher disease in offspring; this reproductive counselling dimension requires specialist genetic counselling expertise beyond the scope of general neurology practice.

Taken together, these considerations lead to a clear conclusion: the PVI framework as currently proposed would be equitable only if implemented within research studies that (a) include diverse populations across ancestral backgrounds and healthcare contexts; (b) establish locally validated reference ranges for all scored components before applying thresholds; (c) provide appropriately trained genetic counsellors for pre-test and post-test counselling in studies involving genetic testing; (d) use culturally validated olfactory testing instruments or alternative measures of olfactory function; (e) actively address access disparities in study design, including by not requiring expensive investigations such as DAT-SPECT or lumbar puncture in all study participants. The PVI must not be implemented in ways that systematically advantage patients with better healthcare access, more favourable ancestry-matched reference data, or greater proximity to specialist academic centres.

## 7. Conclusions

The central message of this review is that the clinical heterogeneity of Parkinson’s disease has a plausible biological basis in selective neuronal vulnerability. Current evidence points to five interacting domains that influence whether a neuron can tolerate alpha-synuclein exposure: calcium handling, mitochondrial and energetic capacity, lysosomal clearance, axonal and synaptic architecture, and the local inflammatory environment. The strongest translational example is the GBA1/GCase pathway, where genetic and enzymatic variation links lysosomal biology to measurable clinical risk.

The Parkinson’s Vulnerability Index proposed here translates this biology into a testable research framework. By integrating genetic, enzymatic, protein-seeding, cognitive, olfactory, and imaging data, the PVI provides a structured research instrument for testing whether vulnerability biology improves prediction of individual clinical trajectory and enhances selection for mechanism-targeted therapeutic trials. The PVI has not yet been validated for clinical use and must not be applied outside a formally approved research protocol.

The most urgent research priorities are prospective validation of the composite score, direct comparison with existing biological staging systems, systematic study of resistant neuronal populations, and redesign of neuroprotective trials around biological vulnerability rather than clinical diagnosis alone. Future studies should test whether adding vulnerability measures to alpha-synuclein status, genetic status and neurodegeneration markers improves prediction sufficiently to justify their use in a trial design.

Precision medicine in PD will depend on matching biological targets to the patient groups most likely to benefit. Vulnerability-based stratification may help move the field in that direction, provided it is tested carefully, communicated honestly and used ethically. The evidence is strong enough to justify validation of this framework, but not yet strong enough to treat it as a clinical guideline.

## Figures and Tables

**Table 2:** Positioning of the proposed Parkinson’s Vulnerability Index relative to established PD staging and biological classification frameworks.

Framework	Primary Anchor	Main Contribution	Relevance to the PVI
**Braak staging**	Regional distribution of Lewy pathology	Provides a neuropathological map of common disease progression.	Useful for anatomical context, but limited for patient-level prediction because it does not explain cell-intrinsic resistance or susceptibility.
**SynNeurGe**	Pathological alpha-synuclein, neurodegeneration and genetic status	Defines PD for research using a three-part biological classification linked to clinical features [8].	The PVI accepts this biological direction but focuses more specifically on vulnerability mechanisms and prognosis.
**NSD-ISS**	Neuronal alpha-synuclein pathology, dopaminergic dysfunction and functional impairment	Stages neuronal alpha-synuclein disease across a biological and clinical continuum [7,9].	The PVI is complementary: it is intended to stratify vulnerability within diagnosed or biologically defined PD rather than replace disease definition.
**PVI proposed in this review**	Vulnerability domains linked to measurable biomarkers	Provides a hypothesis-generating score for prospective validation and trial enrichment.	It is not currently a validated clinical instrument and should be interpreted as a research framework.

Note: This table clarifies the relationship between the PVI and existing validated staging systems, emphasising that the PVI is a complementary research tool, not a competing disease classification framework. Abbreviations: NSD-ISS = Neuronal alpha-Synuclein Disease Integrated Staging System; PD = Parkinson’s disease; PVI = Parkinson’s Vulnerability Index; SynNeurGe = Synuclein–Neurodegeneration–Genetics research diagnostic criteria.

**Table 1:** Evidence profile for the five vulnerability mechanisms reviewed in this article.

Domain	Principal Mechanism	Main Evidence Base	Translational or Clinical Correlate
Calcium handling	Autonomous pacemaking and reliance on calcium channels increase mitochondrial oxidant stress in vulnerable SNpc neurons.	Human regional vulnerability patterns, calbindin sparing, and experimental studies of L-type and T-type calcium channels.	Biologically strong, but the negative STEADY-PD III trial shows that target engagement and patient selection remain unresolved.
Mitochondrial and energetic capacity	Large axonal fields, dopamine handling and impaired Complex I activity create a high energetic burden.	Human SNpc Complex I deficiency, dopamine oxidation studies and experimental work linking arborisation size to vulnerability.	Highly plausible mechanism; direct patient-level biomarkers for energetic vulnerability remain limited.
Lysosomal clearance	Reduced GCase activity and related lysosomal dysfunction impair alpha-synuclein degradation and can create a bidirectional pathogenic loop.	Human genetics, GBA1 cohorts, enzyme activity studies, post-mortem pathology and iPSC-derived dopaminergic neurons.	Strongest translational domain because GBA1 and GCase link mechanism to measurable clinical risk.
Axonal and synaptic architecture	Long, thin, poorly myelinated projections place synaptic terminals at risk when transport, energy supply or local clearance fail.	Post-mortem synaptic pathology, mitochondrial dysfunction at synapses and experimental propagation studies.	Clinically important, but living-patient measures of synaptic vulnerability are still emerging.
Local inflammatory environment	Dense microglial populations in the SNpc amplify alpha-synuclein-triggered cytokine and oxidative responses.	Human single-nucleus studies, microglial activation data and astrocyte reactivity experiments.	Important modifier of vulnerability; causal direction and patient-level inflammatory markers require further validation.

Note: Evidence quality is based on reproducibility across independent platforms, sample sizes, and consistency with human neuropathological data. Mechanisms are ordered from those with the strongest translational biomarker evidence (lysosomal domain) to those with emerging but not yet fully validated patient-level measures (axonal/synaptic and neuroinflammatory domains). Abbreviations: GBA1 = glucocerebrosidase acid beta-1 gene; GCase = glucocerebrosidase enzyme; iPSC = induced pluripotent stem cell; LRRK2 = leucine-rich repeat kinase 2; PD = Parkinson’s disease; PFF = pre-formed fibrils; SNpc = substantia nigra pars compacta; STEADY-PD III = Safety, Tolerability and Efficacy Assessment of Dynacirc CR for PD trial; VTA = ventral tegmental area.

**Table 3:** Proposed Parkinson’s Vulnerability Index (PVI) scoring criteria with published biomarker thresholds. Important notes: (1) All thresholds are provisional and platform-specific; they are not universal cut-offs. (2) Point weights are illustrative candidate values, not validated regression coefficients. (3) Peripheral DBS GCase activity does not directly reflect brain lysosomal GCase function. (4) SAA scoring applies to validated CSF assays only; blood-based or EV-derived SAA platforms are not equivalent. (5) UPSIT norms require local cultural validation. Abbreviations: CSF = cerebrospinal fluid; DAT-SPECT = dopamine transporter SPECT; DBS = dried blood spot; EV = extracellular vesicle; GBA1 = glucocerebrosidase acid beta-1 gene; GCase = glucocerebrosidase; LRRK2 = leucine-rich repeat kinase 2; MoCA = Montreal Cognitive Assessment; SAA = seed amplification assay; SBR = striatal binding ratio; UPSIT = University of Pennsylvania Smell Identification Test; VUS = variant of uncertain significance; WGS = whole-genome sequencing.

What Is Measured	How It Is Measured	Cut-Off Value or Evidence Basis	Illustrative Candidate Weight (Indicative Only; Not Statistically Derived)
**Domain 1: Lysosomal and Genetic (how well the cell clears waste protein)**			
GBA1 gene: high-biological risk pathogenic variant (e.g., L444P; also includes RecNciI, RecA, null variants; heterozygous carriers) (note: the binary high-risk/low-risk classification is a simplification; risk varies with residual enzyme activity, ancestry, and co-occurring modifiers)	Blood: gene test (targeted panel or WGS)	Variant confirmed	3
GBA1 gene: lower biological-risk pathogenic variant (e.g., N370S heterozygote; most prevalent in Ashkenazi Jewish and European populations) (note: penetrance is substantially below 50% and varies by ancestry and co-occurring genetic modifiers)	Blood: gene test	Variant confirmed	2
LRRK2 G2019S gene variant	Blood: gene test	Variant confirmed	2
GBA1 + LRRK2 together	Blood: gene test	Both confirmed	4 (cap)
GCase enzyme activity (CSF)	Lumbar puncture + enzyme assay	<39 nmol/h/mL (Parnetti et al. 2014 platform-specific threshold ONLY; NOT a universal cut-off; local recalibration required before use)	2
GCase enzyme activity (dried blood spot)	Finger-prick card + enzyme assay	<2.8 nmol/h/mg (Lerche et al. 2021 platform-specific threshold ONLY; NOT universal; local recalibration required; peripheral DBS GCase does not directly reflect brain lysosomal function)	1
**Domain 2: Alpha-Syn Pathology (is pathological protein already spreading?)**			
Alpha-syn seeding test (SAA)	Validated CSF seed amplification assay ONLY. Blood-based and EV-derived SAA assays are methodologically distinct and NOT equivalent to CSF SAA without independent platform-specific validation	Positive (validated CSF assay only; blood-based SAA platforms must not be substituted; result is assay-platform specific)	1
**Domain 3: Cognitive Trajectory (is thinking already being affected?)**			
MoCA cognitive score	10 min bedside test	Score < 24 or ≥2-point drop in 12 months	2
Family or carer memory concern	Clinical interview	Corroborated concern present	1
**Domain 4: Olfaction (smell loss is an early PD sign)**			
Smell identification test (UPSIT)	40-item booklet smell test	Below 25th percentile for age/sex	1
**Domain 5: Neuroimaging (dopamine transporter scan)**			
DAT-SPECT dopamine transporter scan	Nuclear imaging scan	Caudate binding <65% norm or asymmetry >20%	1

DBS = dried blood spot; CSF = cerebrospinal fluid; GCase = glucocerebrosidase enzyme; MoCA = Montreal Cognitive Assessment; SAA = seed amplification assay; UPSIT = University of Pennsylvania Smell Identification Test; WGS = whole genome sequencing.

**Table 4:** Prospective Outcome Measures (Module 4)—NOT components of baseline PVI score.

Outcome Measure	How It Is Measured	Definition of Clinically Meaningful Change	Timing of Assessment
Cognitive decline	MoCA score	≥2-point drop from baseline	12 months and annually thereafter
Cognitive concern	Clinical interview	Informant-corroborated concern emerging during follow-up	At each follow-up visit
Motor progression	MDS-UPDRS Part III	≥5-point increase from baseline	12 months and annually thereafter
Conversion to dementia	Clinical criteria	Meets diagnostic criteria for PD dementia	As clinically indicated
Institutionalisation	Clinical record	Placement in long-term care facility	As occurs

**Table 5:** Proposed research monitoring and trial-enrichment implications for PVI risk bands (based on Modules 1–3 only). These suggestions are not clinical guidelines and should be tested prospectively before routine implementation. The risk bands are based on the baseline PVI score calculated from vulnerability biomarkers (Module 1), pathology and staging biomarkers (Module 2), and baseline clinical measures (Module 3). They do not include longitudinal outcome measures (Module 4), which are outcomes to be predicted rather than components of the score.

Area	PVI Low (0–2)	PVI Intermediate (3–5)	PVI High (6+)
Research interpretation	Lower proposed vulnerability; expected slower average progression in validation cohorts.	Intermediate proposed vulnerability; biologically mixed group requiring calibration.	Higher proposed vulnerability; enriched group for progression and dementia-risk studies.
Cognitive follow-up in studies	Annual MoCA or equivalent cohort assessment.	Six-monthly MoCA plus formal neuropsychology if decline is detected.	Three- to six-monthly MoCA within validation protocols; formal neuropsychology if clinically indicated.
Biomarker follow-up	Baseline biomarkers may be sufficient unless phenotype changes.	Repeat GCase or SAA according to study design, especially if score is lysosomal driven.	Repeat GCase, SAA and imaging only within approved research or trial protocols.
Genetic testing and counselling	Genetic testing based on age at onset, family history and local practice.	Testing may be considered with pre-test counselling and consent.	Pre-test and post-test counselling are essential; family cascade testing only where clinically appropriate, consented and locally approved.
Trial enrichment	General neuroprotection or observational cohorts.	Potential enrichment for GCase, lysosomal or alpha-synuclein targeted trials when relevant biomarkers are present.	Potential priority for mechanism-targeted trial screening, not automatic treatment allocation.
Care planning	No PVI-driven ACP requirement.	Routine care planning according to symptoms, patient preference and clinical trajectory.	Planning discussions may be appropriate when cognitive or functional decline is present; score alone is insufficient.
Treatment implications	Standard PD care according to established guidelines.	Standard PD care; additional treatment guided by symptoms and clinician judgement.	Standard PD care; cholinesterase inhibitors, sleep therapies or other additions only when standard diagnostic and clinical criteria are met.

ACP = advance care planning; GCase = glucocerebrosidase; LBD = Lewy body dementia; MoCA = Montreal Cognitive Assessment; SAA = seed amplification assay. IMPORTANT DISCLAIMER: The suggestions in Table 4 are explicitly hypothetical research protocol examples and are NOT evidence-based clinical guidelines. Monitoring intervals and care planning suggestions are NOT supported by direct comparative evidence and must NOT be implemented in clinical practice. The three risk bands (0–2, 3–5, ≥6) are provisional research categories whose clinical utility has not been established. They are included to provide a structured basis for hypothesis-driven validation studies and mechanism-targeted trial enrichment protocols, not as validated prognostic tools. Any investigator wishing to use these bands in a research study must treat them as starting-point hypotheses requiring local recalibration against the specific study population, assay platforms, and pre-specified clinical endpoints.

**Table 6:** Three proposed patterns of PD progression and their possible biological basis. IMPORTANT: The three modes in this table are explicitly hypothetical heuristic illustrations pending prospective validation. The estimated patient proportions (~55-60%, ~20-25%, ~15-20%) are speculative placeholders with no statistical derivation and must NOT be cited as established prevalence estimates. Abbreviations: GBA1 = glucocerebrosidase acid beta-1 gene; LP = Lewy pathology; LRRK2 = leucine-rich repeat kinase 2; PD = Parkinson’s disease.

Mode A: Typical	Mode B: Dementia-Predominant	Mode C: Tremor-Dominant	
Progression	Slow (15–20 years)	Fast: dementia within 3–5 years	Very slow (>20 years)
Dominant vulnerability domain	Mitochondrial and calcium	Lysosomal failure (cortical and nigral)	Partial nigrostriatal impairment; lysosomes largely intact
Likely genetic background	No major variant; sporadic	GBA1 variant; or GBA1 + LRRK2	No major variant; possibly protective modifiers
Cognitive outcome	Dementia late or absent	Early dementia; high cortical LP burden	Cognition largely preserved
Estimated proportion of PD	~55–60%	~20–25%	~15–20%

LP = Lewy pathology; VTA = ventral tegmental area.

## Data Availability

No new data were created or analyzed in this study. Data sharing is not applicable to this article.
